# ‘Two sides of the same coin’? A longitudinal analysis evaluating whether financial austerity accelerated NHS privatisation in England 2013-2020

**DOI:** 10.1136/bmjph-2024-000964

**Published:** 2024-07-16

**Authors:** Benjamin Goodair, Anders Malthe Bach-Mortensen, Aaron Reeves

**Affiliations:** 1 Department of Social Policy and Intervention, University of Oxford, Oxford, UK; 2 Department of Social Sciences and Business, Roskilde University, Roskilde, Sj, Denmark

**Keywords:** Public Health, Patient Harm, trends, economics

## Abstract

**Objectives:**

To understand the relationship between increasing privatisation of the NHS and austerity cuts to public funding.

**Design:**

Longitudinal analysis.

**Setting:**

170 Clinical Commissioning Groups (CCGs) in England between 2013 and 2020.

**Intervention:**

The UK austerity programme, spearheaded by the conservative-led governments of the 2010s, leveraged the 2008 financial crisis to roll-back spending to local government and social security spending. They also restricted the rate of growth in NHS spending—but cuts varied for different areas, often impacting deprived areas hardest.

**Main outcome:**

For-profit outsourcing by NHS commissioners. After the implementation of the 2012 Health and Social Care act commissioners were encouraged and obliged to open contracts to the private sector. The uptake of for-profit outsourcing varied massively. Some CCGs contracted out almost half of their activity, and others almost none.

**Results:**

We calculate the size of austerity across all CCGs. The financial restrictions meant that commissioners had, on average, £21.2 m more debt by 2021 than in 2014 in real terms. We find that there is a null and very small effect of changes to local NHS funding on for-profit outsourcing. A decrease in £100 per capita of NHS funding corresponds in a decrease in 0.441 percentage points (95% CI −0.240 to 1.121) of for-profit expenditure. We also find that local changes to public expenditure on the NHS, local government and social security do not confound the relationship between for-profit outsourcing and treatable mortality rates.

**Conclusions:**

NHS privatisation at the local level does not appear to be a direct response to or result of austerity. That does not mean that it is unproblematic. Rather than being confounded by funding levels, the deteriorating health outcomes associated with privatisation should be considered as a distinct concern to the disastrous health effects of austerity policies.

**WHAT IS ALREADY KNOWN ON THIS TOPIC**

- The NHS in England has experienced an increase in outsourced treatments and expenditure on for-profit providers of healthcare over the last 10 years. Meanwhile, levels of funding for the NHS have grown in absolute terms, but has not followed demand, and the relative increases have slowed since 2010. Both of these trends (outsourcing and underfunding) have been linked with additional mortalities.

**WHAT THIS STUDY ADDS**

- Our results show that changes to local levels of funding are not associated with local levels of outsourcing in the following year—suggesting that outsourcing is not, on average, a direct response to funding shortfalls. We know that the associations between outsourcing and increased mortality are not confounded or moderated by the relative levels of funding at the local level.

**HOW THIS STUDY MIGHT AFFECT RESEARCH, PRACTICE OR POLICY**

- The implications of this study primarily relate to the question of whether the impact of outsourcing NHS services to for-profit companies on quality of care is explained by funding cuts. We directly test if outsourcing is a result of underfunding, and whether impacts on health outcomes are therefore primarily the result of austerity rather than provider ownership. Given the lack of association, this analysis suggests that the impacts of for-profit outsourcing are due to the impact of increased for-profit ownership itself.

## Introduction

 The Conservative Party in the UK, which was in power between 2010 and 2024, sought to significantly reduce government spending to address budget deficits generated by the 2008 financial crisis. Reducing funding was intended to compel existing service providers to become more efficient by delivering certain tasks with a smaller budget and by cutting back areas of support with little added value.^1^ At the same time, health and social care commissioners could seek to maintain service provision while paying providers less. In practice, this meant privatising state-funded services by outsourcing to for-profit providers.^2^ The supposition was that allowing the private sector to compete with the NHS would improve (or at least maintain) quality while reducing costs to the taxpayer.^2^,^4^ To facilitate this, the government expanded the private sectors access to NHS contracts via the 2012 Health and Social Care Act and by limiting growth in NHS funding. In hindsight, it has become clear that the space for efficiency gains seems smaller than the government imagined at the time, and the quality of public services appears to have worsened in the years since.^5^,^7^ However, what remains unclear is whether funding cuts did, in fact, accelerate privatisation, and whether commissioners used privatisation as a tool to adapt to austerity.

This puzzle is significant because it potentially helps us understand stagnating life expectancy since 2011. Both austerity and privatisation have been evidenced to correspond with declining quality of care and worsening health outcomes.^8^,^10^ If austerity and privatisation are related, the association between privatisation and health could be entirely confounded by levels of funding, and we might conclude that private provision is not itself the cause of worsening care. Alternatively, it could be that privatisation connects the causal pathway between austerity and health outcomes or, finally, that these events happen independently of each other. Unpacking these associations is key to understand whether, and if so, how, austerity impacts health and how privatisation impacts health.

### Retrenching the state: the privatisation-austerity nexus of the 2010s

The policy debate surrounding healthcare privatisation is a long-standing and contentious topic in England. Consecutive governments have implemented reforms to, step-by-step, introduce private sector provision into the NHS. Thatcher’s 1990 marketising reforms were part of a concerted long-term privatisation plan that aimed to make the NHS more ‘business-like’ and responsive to ‘customer’ needs.^11^ The moves in the 1990s towards an increasingly marketised provision of the NHS resulted in a significant academic debate about the impacts of competition on the quality of care, with mixed conclusions.^12^,^14^ Then, New Labour’s privatisation—via the agreements with ‘Independent Sector Treatment Centres’ (ISTCs)—were framed in terms of target- and performance-driven policymaking which, for the NHS, often centred on waiting-times: ‘*in order to sustain lower waiting times while continuing to treat patients according to clinical need, a permanent structural increase in the volume of healthcare services delivered to patients would be required.’*^15^ The introduction of ISTCs in the 2000s has been evaluated in relation to the impact of private sector competition on health service performance, with some studies finding shortening length of stays or higher treatment volumes in areas that had private sector competition.^16 17^

The incoming conservative-led coalition government in 2010 leveraged the 2008 financial crisis to justify many of its reforms, blaming a need for reducing public debt despite these being ideologically informed political decisions, including reforms that were designed to outsource NHS services.^18^ Repeating the previous political rhetoric of making the NHS more responsive to consumers and improving performance, a new argument was introduced for outsourcing services: that this enabled the NHS to adapt to the ‘necessary’ cuts to public funding. For example, in a speech about the NHS, David Cameron stated: ‘*we need to make the supply of healthcare more efficient - which is why we are opening up the system to new providers*…’.^19^ The potency of this political paradigm rationale was evident, even in local NHS documents.^20^

In the 2010s, privatisation and austerity were a political nexus, and the narrative designed in a way that enabled politicians to use both concepts to legitimise and reinforce one another. The prevalent political discourse justified increasing outsourcing through the necessities of austerity. Meanwhile, austerity was enabled by the financialisation, commercialisation and deregulation of certain public services.^21 22^ Privatisation and austerity were implemented in the 2010s with a shared objective: to retrench the state. The burden of the financial crisis was outsourced, both in terms of shifting the burden to individual families receiving lower social security payments, but also in terms of outsourcing public services to private companies willing to implement budget costs and service reductions that ultimately came at the expense of service quality.

### How would austerity increase NHS privatisation locally?

There are two possible reasons why NHS commissioners may respond to government-imposed austerity through privatisation. (1) Areas with worse financial resources are under the most pressure to cut costs by politicians and commissioners. In a push to find ‘efficiencies’ in delivering the same level of healthcare, commissioners have been encouraged to turn to the market.^23 24^ Through outsourcing processes, competition is supposed to enable them to find providers who will deliver the same services for less cost. (2) Austerity increases the need—but not the support—for healthcare.^25^ Lower levels of public funding and worsening social determinants of health since 2010 have been strongly linked to worse population health in England.^6 8 10^ With evidence linking austerity to a range of worsening mortality, health and life expectancy are measured.^26^,^29^ And, with devolved governments having their budgets cut by Westminster, the negative impact of austerity on health has been recorded in all nations of the UK.^30^,^32^ The cuts to welfare and local authority services may then drive privatisation via increased demand on already constrained NHS services, which, in turn, may force commissioners to supplement existing provision with private services.

Commissioner decision-making around outsourcing is informed by a range of relational, political and informational determinants.^24 33^ Some commissioners feel they have flexibility in deciding who provides NHS services, while others feel that the legislation constraints their freedom to decide.^33^ Commissioners certainly mention financial pressures as one of the reasons they might turn to formal procurement processes from the market.^23^ Similarly important are the availability of effective private provision in their geographic areas, the strategic/political direction of local commissioning leadership, and the need of local populations.^24^ Financial constraints might not, therefore, be the sole (or even primary) reason for increased outsourcing.

Why would some commissioners feel financial pressure more than others during this period? The NHS allocation was intentionally distributed to equalise health inequalities under New Labour’s time in power, with some evidence of successful results.^34^ But this tilt towards deprived areas changed in the early 2010s under the conservative-led coalition with new directives towards finding efficiencies, and early evidence suggests a slowing of equality improvements.^35 36^

### Is an association between austerity and privatisation important?

The policy implications of the association (or the absence of one) between austerity and privatisation are significant. Below, we briefly discuss the implications of the existence and absence of this relationship through the three scenarios, which will be explored and tested in this paper. If austerity is associated with higher privatisation, we might be observing the intended implementation of privatisation policies by the conservative government. Outsourcing is used by the commissioners, who are experiencing the most financial pressure. This would help explain why levels of privatisation vary substantially among areas. Unpacking this relationship will enhance our understanding of the impacts of NHS privatisation. Evidence suggests that privatisation corresponds to higher mortality rates in areas with the most for-profit outsourcing.^9 37^ The conclusion of these studies is that, on average, privatisation is associated with worse quality of care for service users.^38^ However, the causal pathway through which privatisation impacts quality of care is unclear and contested, especially given the observed null differences in outcomes between public and private hospitals in England. This means that it is an open question whether private provision is of worse quality.^39 40^ If austerity drives an increase in privatisation, it suggests that the aggregate associations between privatisation and health outcomes are not representative of the impacts of increased for-profit healthcare and that it is in fact driven by austerity cuts. More technically, the conclusion would be that privatisation and its relationship on health are confounded by austerity cuts. In evaluating the promise of pro-market reforms going forward, it is therefore of key importance to evaluate the extent to which outsourcing relates to austerity, and whether the effect of privatisation is independent or explained by shifts in funding.

**Figure 1:**
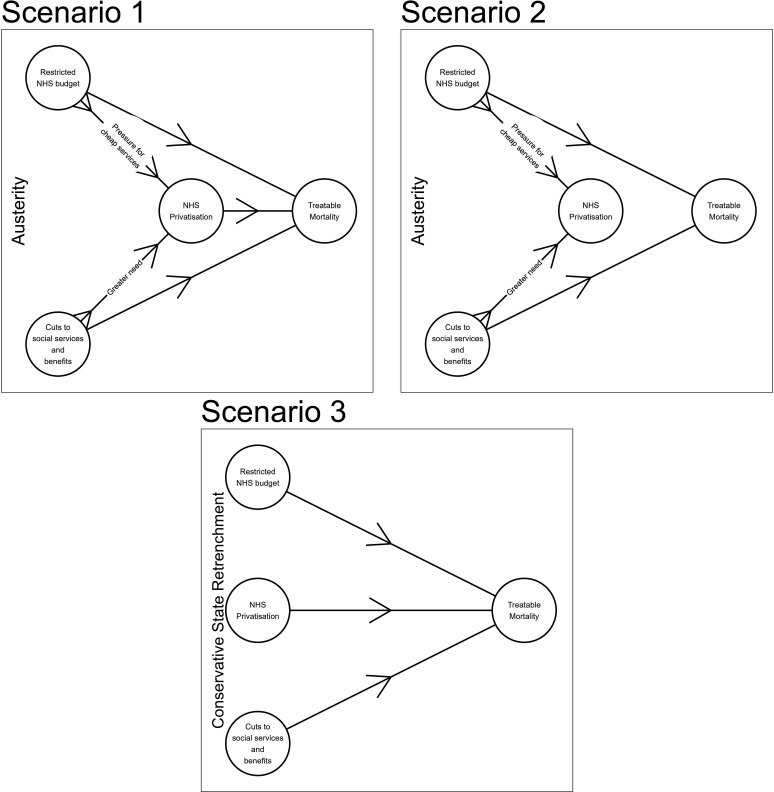
Diagram of potential causal pathways.

Figure 1 details the three potential causal pathways, which might be occurring. In scenario 1, austerity causes increased privatisation, and mortality rates are impacted by both privatisation and austerity. In scenario 2, the relationship between privatisation and health outcomes is confounded by the levels of government funding, so that once accounting for austerity, the relationship between privatisation and health would disappear, thus suggesting that privatisation itself is not reducing the quality of care. In scenario 3, austerity and privatisation are not related, and each has their own independent relationships with health outcomes.

## Methods

### Data

This paper constructs three novel datasets detailing the levels of austerity between 2010 and 2020 over unprecedented time periods and at the scale of health geographies, using allocated budgets from central government for NHS commissioners, local authorities and welfare benefits. Our independent variables of interest are the per-capita constant prices funding of NHS commissioners, local authorities and welfare benefits (universal credit and the components it replaced). Note that the NHS and local authority variables represent funding allocations, whereas the social security variable is the actual expenditure value (as funding levels are the same given the level of need). All data is built up to the NHS commissioner’s geographic scale using best fit lookups where required.^41^

The paper also uses a database of monthly expenditure files, curated between 2013 and 2020 for each NHS commissioner for which the data was available.^42^ The data builds on work done to make procurement data from individual government bodies widely available.^43 44^ This database allows us to track the percent of expenditures being spent on for-profit companies by local healthcare commissioners, which is the measure of privatisation in this analysis. During this period GPs were primarily funded directly by NHS England—meaning that most of the local Clinical Commissioning Group (CCG) expenditure will exclude primary care—and rather be comprised of secondary and community care services.

The paper uses local mortality rates of amenable causes from the ONS.^45^ The measures used in this paper are the age-standardised rates of death per 100 000 in the population, aggregated to the CCG area scale. We primarily use rates of treatable mortality. Treatable mortality is defined as ‘causes of death that can be mainly avoided through timely and effective healthcare interventions’ (ibid.). For example, treatable mortalities include causes such as respiratory diseases, certain types of cancer like Hodgkin’s disease, and ‘misadventures’ to patients during care. We may expect healthcare privatisation and healthcare funding to primarily impact treatable mortality through declining quality of healthcare interventions, whereas other forms of austerity may be more likely to impact preventable mortality through worsening social determinants of health. It is for this reason that we conduct our analyses on all three measures.

The analyses are conducted on all 170 clinical commissioning groups for which expenditure data is available in England over a 7 year period, 2013–2020. A full description of the data building process, missing data and potential limitations can be found in online supplemental appendix A1 . Raw data come from a range of publicly available sources.^46^,^50^ All harmonised data and code to reproduce the analyses, in their entirety, are published openly at https://github.com/BenGoodair/austerity_and_privatisation and in a minted repository.^51^

### Analysis

The paper performs two stages of analysis. First, the paper analyses whether there are longitudinal area associations between the independent variable of levels of austerity and dependent variable of levels of NHS privatisation. We conduct two-way fixed effects regressions, controlling for population size. The analysis is intended to understand whether increases in for-profit outsourcing correspond with the varied decreases in public funding over time (scenarios 1 and 2 in figure 1).

For the next stages of the analysis, we recreate the linear two-way fixed effects regressions from Goodair and Reeves, which found that increases in privatisation correspond with increases in treatable mortality. We test whether this relationship is confounded by austerity, and we systematically control for austerity related to the NHS, LA and benefits. We also assess whether the relationship between privatisation and mortality is moderated by austerity by using interaction effects (see online supplemental appendix A2). These analyses are designed to test the association between privatisation and mortality in the scenarios in figure 1, and whether the connection between privatisation and mortality might be moderated by austerity (scenario 3).

This paper has two key theoretical estimands^52^:

1. The changes to local expenditure on private providers realised in one geographic area given the changes in the levels of government funding.
2. The changes to the statistical association between increased privatisation and increased mortality given changes in levels of government funding.

### Robustness checks

We perform several robustness checks, including using alternative measures of austerity, privatisation and mortality. First, we run our models on changes to the reported end-of-year balance in CCG accounts, as these might better identify the experience of restricted budgets rather than the impact of central government decision-making on allocations per se (online supplemental appendix A3). We also conduct the analyses using the percent of acute treatments delivered by independent providers instead of the expenditure on for-profit companies, as this measure might better identify the pathways indicated by increased need (online supplemental appendix A4). Finally, we run the regressions with ‘avoidable mortality’ as an alternative outcome measure. This is a measure of mortality that includes causes with public health solutions such as lung cancer, sexually transmitted diseases and traffic incidents (a full list of the treatable and preventable causes of mortality is available in online supplemental appendix A.1.6). This measure might better capture the deaths from causes linked to deteriorating social determinants of health (online supplemental appendix A5).

### Patient involvement

No patients were involved in setting the research question or the outcome measures, nor were they involved in developing plans for design or implementation of the study. No patients were asked to advise on the interpretation or writing up of results. There are no plans to disseminate the results of the research to study participants or the relevant patient community.

## Results

**Figure 2:**
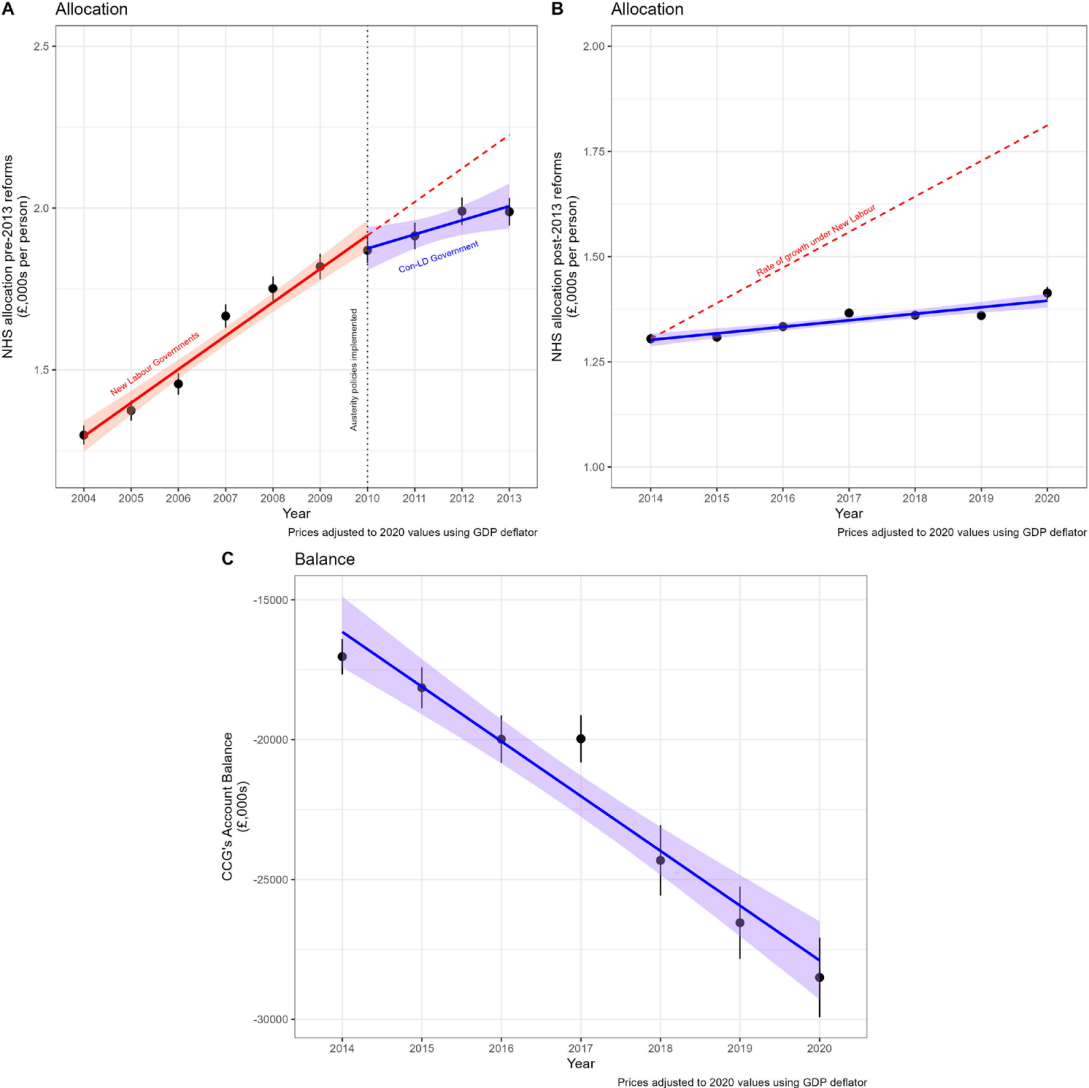
Impacts of austerity period on NHS allocations. Panel A depicts the absolute allocation amounts between 2003–2004 and 2012–2013, the undashed lines represent the line of best fit before and after austerity programme was introduced in 2010, and the dashed line represents the continued rate of growth before 2010. Panel B depicts the allocation amounts after major NHS reforms, which changed the responsibility of commissioners, making their allocation incomparable, and the dashed red line represents the annual 6% growth observed in our data before 2010. Panel C shows the end-of-year balance reported in Clinical Commissioning Group accounts.

Figure 2 shows the level of funding allocated to NHS commissioners and their reported end-of-year accounts balance. It shows that funding had real terms increases after 2010, but that the increases were much lower than in the previous period. For the NHS, ‘austerity cuts’ represented a reduction to the funding increases, which did not match the increasing need for the NHS.^53^ We also see that this change happened immediately after 2010 (see panel A). Panel B shows the change to allocations after the commissioning bodies were reformed in 2013 (and with it a change to responsibilities) and also plots what the allocation would have looked like had the pre-2010 growth rate continued at 6%. Financial restrictions seen since 2010 meant that commissioners had, on average, £21.2 m more debt by 2021 than in 2014 in real terms (panel C). See online supplemental appendix A6 for the real terms reductions in the levels of government expenditure on local authority funding and social security spending.

Even though there were real terms increases in funding, the reduced NHS growth has been reported to impact the accessibility of NHS services in England through strategies of delay, denial, deflection, selection, deterrence and dilution.^54^ Meanwhile the reduced growth has also corresponded with increases to avoidable mortality rates.^10 55^ Our data finds some variation in whether different NHS commissioners received increases in funding since reforms to the organisations in 2013–2014. For example, a handful of London-based CCGs had real terms cuts like Tower Hamlets, Camden and Central London Westminster. Camden had a £1550 per capita in 2014, which had reduced to £1370 by 2019 (all prices are adjusted to 2020 prices). Conversely, Bradford City, which started on a similar £1530 in 2014, saw an increase to £1790 by 2019. See online supplemental appendix A7 for a full description of funding levels by CCG.

**Figure 3:**
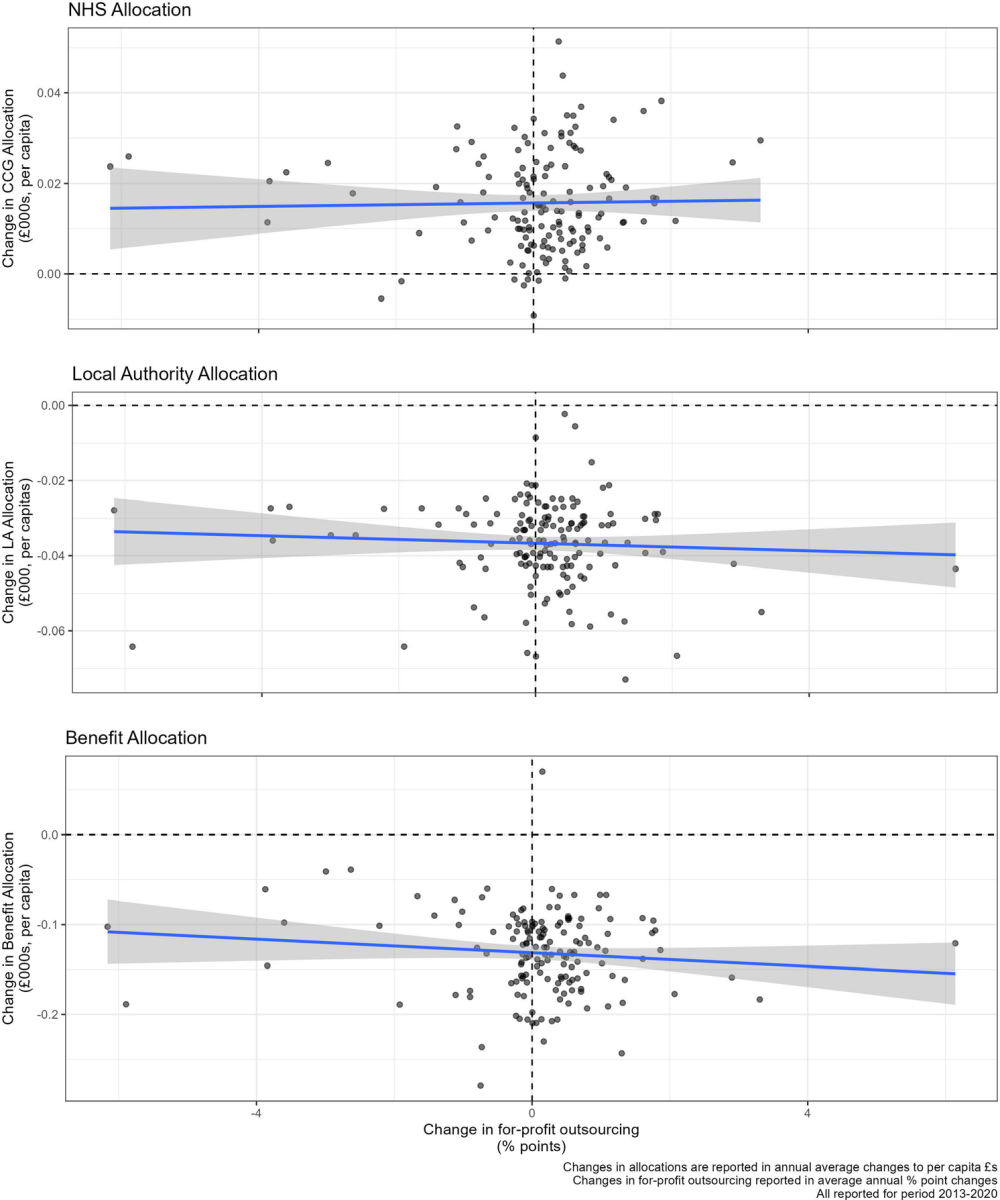
Relationship between austerity and privatisation.

Figure 3 plots the variation in changes to funding levels against the changes in levels of privatisation in those areas. Along the x-axis is the increases in for-profit expenditure by NHS commissioners between 2014 and 2020, and the y-axis reports changes to different types of government funding. It shows that there is little relationship between austerity and privatisation geographically. The areas with the biggest increases in privatisation are not the same places most impacted by austerity cuts in terms of real changes to funding. The figure also visualises the severe cuts to local authority and welfare benefits over this time period.

Table 1 assesses the same associations as in figure 3, using panel regression models controlling for the population size and total CCG expenditure. The results in table 1 show that a decrease in £1000 per capita of NHS funding corresponds in a decrease in 4.41% points (95% CI −2.40 to 11.21) of for-profit expenditure. This is a null finding, with large CIs, suggesting that the relationship is not strong enough, the sample size not large enough or the variation too wide to identify a meaningful relationship between austerity and privatisation. The effect size is also small. An increase in £1000 per capita would represent a doubling of NHS allocation for many CCGs, but this would not correspond with a radical change in the % of for-profit outsourcing. Results are similar for the allocation of nearby local authorities and expenditure on benefits.

**Table 1:** Association between austerity and privatisation

	For-profit outsourcing(% points) (.95 CI)	P value	For-profit outsourcing(% points) (.95 CI)	P value	For-profit outsourcing(% points) (.95 CI)	P value
CCG allocation (£000 s per capita)	4.4060 (-2.4012, 11.2133)	0.3488				
Local authority allocation (£000 s per capita)			−3.4317 (-9.0118, 2.1484)	0.3003		
Benefit allocation (£000 s per capita)					5.4118 (-16.1126, 26.9362)	0.6818
Num.Obs.	608	608	785	785	784	784
R2	0.002	0.002	0.002	0.002	0.005	0.005
R2 Adj.	−0.374	−0.374	−0.291	−0.291	−0.288	−0.288
CCG fixed effects	Yes	Yes	Yes	Yes	Yes	Yes
Time fixed effects	Yes	Yes	Yes	Yes	Yes	Yes
Clustered standard errors	Yes	Yes	Yes	Yes	Yes	Yes
Control variables	Yes	Yes	Yes	Yes	Yes	Yes

Results from multivariate longitudinal regression models. Robust Ses are clustered at Clinical Commissioning Group level and use a bias-reduced linearisation estimator (CR2). Lag of 1 year applied to allocation variables, which are also log transformed. Control variables are total commissioner spend and population size.

CCGClinical Commissioning Groups

Table 2 tests whether the results from Goodair and Reeves^9^ that for-profit outsourcing corresponds with higher mortality rate is confounded by government austerity by controlling for funding levels. When controlling for CCG funding, LA funding and benefit spend, the effect size between privatisation and mortality rate remains the same and the statistical significance of this relationship increases (becomes even more significant with lower p value and narrower CIs). For the full regression models, see online supplemental appendix A8.

**Table 2:** Assessment of potential confounding in the association between privatisation and mortality

	**Ln.** treatable mortality (.95 CI)[P value]	Ln. treatable mortality (.95 CI)[P value]	Ln. treatable mortality (.95 ci)[P value]	Ln. treatable mortality (.95 ci)[P value]	Ln. treatable mortality (.95 ci)[P value]
For-profit outsourcing (%)	0.0039 (0.0020, 0.0058)[0.0058]	0.0038 (0.0020, 0.0056)[0.0052]	0.0039 (0.0019, 0.0058)[0.0064]	0.0040 (0.0021, 0.0058)[0.0045]	0.0039 (0.0021, 0.0057)[0.0048]
CCG allocation (£000 s per capita)		−0.1964 (-0.5349, 0.1421)[0.2625]			−0.2085 (-0.5565, 0.1396)[0.2475]
Local Authority allocation (£000 s per capita)			0.0514 (-0.2411, 0.3439)[0.7328]		0.1350 (-0.1816, 0.4517)[0.4097]
Benefit allocation (£000 s per capita)				−0.0460 (-0.1220, 0.0300)[0.2649]	−0.0521 (-0.1326, 0.0285)[0.2412]
Num.Obs.	502	484	502	502	484
R2	0.049	0.049	0.049	0.051	0.052
R2 Adj.	−0.357	−0.359	−0.361	−0.358	−0.362
CCG fixed effects	Yes	Yes	Yes	Yes	Yes
Time fixed effects	Yes	Yes	Yes	Yes	Yes
Clustered standard errors	Yes	Yes	Yes	Yes	Yes
Control variables	Yes	Yes	Yes	Yes	Yes

Table reports results from multivariate longitudinal regression models. Robust Ses are clustered at Clinical Commissioning Group level and use a bias-reduced linearisation estimator (CR2). Lag of 1 year applied to allocation variables. Tr. Mortality and allocation variables are log transformed, ‘Ln’ denotes the natural log of outcome variable. Control variables are household income, total commissioner spend and population size.

Online supplemental appendix A2 presents results from similar analyses to see if the relationship between privatisation and mortality is moderated by levels of government funding. Similarly, we find no statistically significant relationship for the interaction between austerity and privatisation. The relationship between privatisation and mortality rates does not vary according to the experiences of austerity.

## Discussion

The results of this paper suggest there was no geographic association between the level of public funding and NHS outsourcing. Moreover, our results suggest that the relationship between outsourcing and mortality appears to be distinct from the corresponding levels of austerity. This adds evidence towards a growing body of evidence that privatisation policies caused additional harm to service users, rather than alleviate it. This has several large implications for our understanding of how privatisation functions and its potential impacts on the quality of care.

NHS privatisation in England is associated with more people dying of treatable causes locally; a trend that has coincided with government austerity programmes that have since been coined as the ‘lost decade’ or ‘the decade that broke Britain’.^56 57^ Cuts to public services and benefits are widely evidenced to cause worse health outcomes for the population.^26^ Privatisation was meant to alleviate this harm by enabling commissioners to uphold quality at reduced costs. The evidence in this paper suggests that not only are austerity and privatisation unrelated but also privatisation seems to have directly and independently exacerbated mortality outcomes.

The pathways to harm related to austerity cuts are well documented.^5 26 27^ But there is less research that explains how privatisation can be thought to cause poorer healthcare outcomes. Opponents of privatisation suggest that this is explained by (a) quality differentials between for-profit and public providers, meaning that privatisation simply introduces more poorly performing providers into the NHS and or (b) ‘knock on effects’ of privatisation—that outsourcing services might impact on the ability of public hospitals to maintain quality standard given changes to their income and patient case-mix.^58 59^ Further research is urgently needed to tease out these mechanisms empirically and evidence the causal pathway from privatisation to deteriorating patient health.

These findings are timely as the incoming Labour Government is planning further privatisation of services.^60 61^ The absence of a relationship between funding levels and outsourcing is particularly noteworthy, in that it questions the potential of privatisation as a tool to improve the quality and costs, even if more money was available to healthcare commissioners.

**Figure 4:**
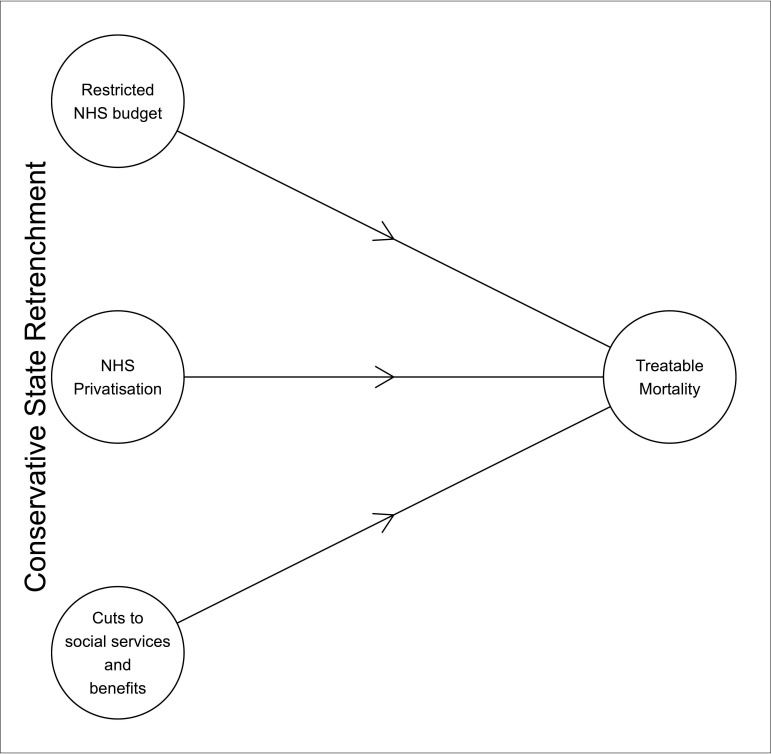
Diagram of suggested causal pathway following the investigation.

Figure 4 describes the consequential causal pathways suggested by our investigation. Privatisation, NHS spending cuts and cuts to social services and benefits are all associated with higher levels of amenable mortality at the local level. Our results help understand this association, by documenting that they appear to do so independently of each other.

The intention of this paper is not to suggest that the two policies are unrelated. Politically, they have been introduced as connected policies that are both designed to retrench the state. Importantly, it was the ‘austerity politics’ of the 2010s—as opposed to financial austerity—which enabled policies such as those which increased NHS privatisation.^56^ Their common political roots mean that we can and should scrutinise potential joint effects. Our results indicate that each policy has contributed independently to the deterioration of the NHS and that privatisation was not systematically associated with austerity cuts.

One limitation of this study is that the relationship might not be valid for other services over this period. For example, the null relationships may not hold for other services like social care, for which the cuts to the commissioners (local authorities) have been much more severe and the increases in outsourcing much higher.^62^,^64^ The more regulated healthcare sector in England may have protected the sector against inverse provider incentives, compared with other public services, even if we still see associations with worse health outcomes. It should also be noted that the associations between higher levels of for-profit outsourcing and higher mortality rates at an ecological level do not necessarily imply that private provision is of lower quality due to the aggregate level of analysis and the use of averages in producing this finding. Indeed, much of the evidence comparing quality in private versus public hospitals in England often finds null differences in health outcomes.^39 40 65^

The methods available to researchers studying the impacts of austerity and privatisation are often insufficient to establish a causal relationship due to the lack of experimental settings and the potential for residual confounding. Instead, the aim of this paper was to identify the relationships we might expect to observe were there was a true causal relationship. In the absence of a relationship between local allocations and local outsourcing, we can build evidence towards, but not be certain of, a causal understanding of these two important features of the health system.

One unanswered question remains: if privatisation has not been implemented because NHS commissioners were reacting to budget cuts, does that mean that services in the private sector are cheaper? Previous research find little differences in the productivity of public and private hospitals.^66 67^ However, further research should test whether the privatisation of services results in cheaper services on aggregates. Such a work should carefully consider differences in the type of services and patient characteristics to account for treatment and user complexity. Speculatively, the reasons we do not observe financial cuts corresponding with further outsourcing could be because commissioners cannot find cheaper provision from for-profit providers or because commissioners are simply more or less predisposed to outsource irrespective of the cost constraints they face at the CCG level.

## Conclusion

It is inherently difficult to analyse the impact of public service reforms in England in the 2010s distinctly from the underlying austerity programme in which they were implemented. The same goes for NHS privatisation, which was introduced in an attempt to cut costs in healthcare without compromising quality. This paper is the first to test whether the impacts of NHS privatisation are dependent on levels of government funding. In finding that funding levels largely do not impact privatisation, our results suggest that the impacts of privatisation are likely independent and thus serve as an additional source of harm on top of that caused by austerity. Consequently, privatisation and austerity cuts should be treated as distinct concerns to the disastrous developments experienced by the NHS.

The results of this paper have important implications for the continued privatisation of healthcare provision in England. In a scenario in which fiscal austerity is alleviated, it might be expected that privatisation would not harm healthcare quality. The findings in this paper suggest that the privatisation of healthcare services has similarly poor effects on health outcomes, regardless of the fiscal environment.

## supplementary material

10.1136/bmjph-2024-000964 online supplemental file 1

## Data Availability

Data are available in a public, open access repository.
