# Supplementary material for: ‘Two sides of the same coin’? A longitudinal analysis evaluating whether financial austerity accelerated NHS privatisation in England 2013-2020
Source: BMJ Public Health. 2024 Jul 16;2(1):e000964. doi: 10.1136/bmjph-2024-000964 (PMC11812912; doi:10.1136/bmjph-2024-000964)
Supplement: online supplemental file 1 [file bmjph-2-1-s001.pdf]

Supplementary Material

Contents

Supplementary Material ... 1

Data ... 2

A.1 – Data cleaning ... 2

A.1.1 – Privatisation ... 2

A.1.2 – CCG funding ... 2

A.1.3 – LA allocation ... 3

A.1.4 – Benefits expenditure ... 3

A.1.5 – Mortality rates ... 3

A.1.6 – Causes of avoidable mortality ... 3

A.7 – Data descriptives ... 8

A.7.1 – summary table ... 8

A.7.2 – summary by CCG ... 8

A.7.3 – summary table by year ... 18

A.6 – Data visualisation ... 19

A.6.1 – LA Allocation ... 19

A.6.2 Benefits expenditure ... 20

Supplementary Analyses ... 20

A.8 Confounding privatisation ... 20

A.2 Moderating privatisation ... 21

A.5 Confounding privatisation on avoidable mortality ... 21

A.3 CCG Balance ... 22

A.4 Private sector treatments ... 23

A.9. Data breakdowns ... 24

A.10. Test for Multicollinearity ... 25

References ... 25

This appendix is ordered to flow from a) data cleaning to b) data description to c) supplementary analyses with results to d) robustness checks. The appendix is labelled in order of the appearance in the main manuscript.

Data

A.1 – Data cleaning

This research contains several novel aggregated datasets. They have been aggregated in a mixture of computationally intensive ways. This section will walk through, data source by data source, the processes involved in collecting and cleaning the data – as well as the decisions made and limitations of each measure.

A.1.1 – Privatisation

Privatisation is conceptualised, for the most part, in this research as the % of NHS treatments delivered by private sector providers. We use two measures of this – 1) the % of expenditure from CCGs going to for-profit providers and 2) the % of clinical-led treatments conducting in private providers.

The benefits of the first measure are that it will include all services, not just a subset of treatments. The data also starts in 2013, rather than the end of 2015. And this measure can focus on for-profit providers only – capturing the profit-motivated health providers as opposed to a mixture of for-profit and non-profit companies.

The benefits of the second measure are that it might better capture the pathway from increased health needs to privatisation given that it will solely measure the number of private sector treatments.

A.1.1.1 – CCG expenditure

CCG expenditure is cleaned from the data resource *NHS Spend* (1). Rahal and colleagues scraped monthly expenditure files for all CCGs which published parsable expenditure files (2). They then used algorithmic matching to link this data to companies house and charities commission records – identifying whether the expenditure goes to a public body, for-profit company or registered charity.

We utilise this resource, aggregating the data annually for each available CCG. We divide all expenditure on for-profit companies by the total expenditure to produce our key variable for % privatisation.

A.1.1.2 – CCG treatments

NHS England publishes the waiting times for all acute treatments conducted by the NHS (3). Since October 2015, this data resource has linked the accountable commissioner to the provider. This data includes the total number of treatments conducted monthly by each provider and indicates which provider is an NHS Trust or non-NHS provider.

We take this data, aggregate in annually and calculate the % of treatments each CCG is responsible for conducted in non-NHS providers. We include all treatments, both admitted and non-admitted patients.

A.1.2 – CCG funding

We want to measure the effects of austerity in the NHS. To capture this we measure the levels of funding each CCG receives annually (4). We also include a slightly different measure which will measure experiences of financial difficulty of CCGs – not necessarily corresponding with austerity policies.

The benefits of using the measure of funding is that it accurately represents the allocation from central funding sources – the first manifestation of austerity policies. It is also complete data and was collected manually to ensure accuracy. Regional allocations are, however, determined by algorithms based on local data and anticipated need. Therefore, changes in allocation levels are sometimes a product of demographic changes and the potential confounding should be considered when regressing against health outcomes.

The balance reported by CCGs end of year accounts might provide us with a better measure of the experience of financial difficulty of CCGs (5). This allows us to answer a slightly different question, not

whether austerity drives privatisation, but whether financial difficulty drives privatisation. The downside of this measure is that the causes of a low account balance may be features of accounting rather than actually representative of the CCG needing to find spending cuts. Another downside is that NHS England stopped presenting aggregated commissioner accounts since 2017, so the final years were scraped from annual reports of all CCGs.

A.1.2.1 – CCG allocation

The total allocation provided to CCGs is reported by NHS England annually (4). We take this and aggregate the data to give us a longitudinal value for CCG allocation between 2013 and 2019. We also use best-fit look ups to assess the levels before NHS reforms in 2013 for the visualisation in figure 1 (6).

Annual values for the CCG allocation are then divided by the CCG-s mid-year population estimates – provided by the ONS. And constant prices are calculated using a GDP deflator (7).

A.1.2.2 – CCG accounts balance

CCG annual accounts were published annually in a central location between 2013 and 2017 (5). We take the end of year balance to represent the annual financial health of the CCG. For the years after 2017, we downloaded every CCG end of year report, processed the pdfs in R and parsed rows reporting end of year balance.

A.1.3 – LA allocation

Local authority allocation is calculated as the central government grants given to LAs. This excludes tax revenue and follows the methods of Alexiou and colleagues (8). We take the revenue support grant, and the redistributed non-domestic rates/ retained income from Rate Retention Scheme. These are summed, divided by estimated mid-year population to represent the generosity of central government towards Local Authorities.

A.1.4 – Benefits expenditure

For benefits data, we take the Universal Credit reported by the Department for Work and Pensions (9). We also combine this with all the principal components which the UC replaced (housing benefit, personal independence payments, incapacity support payments, jobseekers allowance, disability living allowance, tax credits, employment and support allowance, we also included carers support allowance). This process was inspired by the work of Beatty and Fothergill who calculated the anticipated regional variation in welfare cuts – to our knowledge this is the first time it has been actually calculated (10).

A.1.5 – Mortality rates

We take the avoidable mortality rates presented at the approximate CCG boundary geographies (11). CCGs do not function on strict geographies but rather based on GP registrations within a certain boundary. Therefore, these represent approximate populations. Avoidable mortality is constructed as an aggregate of two different types of amenable mortality: treatable and preventable. Treatable mortality represents deaths from causes which are medically curable, preventable mortality represents deaths which have a more social/ public health link. The exact causes of death are reported below in A1.6.

A.1.6 – Causes of avoidable mortality

**Condition group and cause**	**ICD-10 codes**	**Age**	**Treatable**	**Preventable**
**Infectious diseases**				
Intestinal diseases	A00-A09	0-74		•
Diphtheria, Tetanus, Poliomyelitis	A35, A36, A80	0-74		•
Whooping cough	A37	0-		•

		74		
Meningococcal infection	A39	0-74		•
Sepsis due to streptococcus pneumonia and sepsis due to haemophilus influenzae	A40.3, A41.3	0-74		•
Haemophilus influenza infections	A49.2	0-74		•
Sexually transmitted infections (except HIV/AIDS)	A50-A60, A63, A64	0-74		•
Varicella	B01	0-74		•
Measles	B05	0-74		•
Rubella	B06	0-74		•
Viral Hepatitis	B15-B19	0-74		•
HIV/AIDS	B20-B24	0-74		•
Malaria	B50-B54	0-74		•
Haemophilus and pneumococcal meningitis	G00.0, G00.1	0-74		•
Tuberculosis	A15-A19, B90, J65	0-74	• (50%)	• (50%)
Scarlet fever	A38	0-74	•	
Sepsis	A40 (excl. A40.3), A41 (excl. A41.3)	0-74	•	
Cellulitis	A46, L03	0-74	•	
Legionnaires disease	A48.1	0-74	•	
Streptococcal and enterococci infection	A49.1	0-74	•	
Other meningitis	G00.2, G00.3, G00.8, G00.9	0-74	•	
Meningitis due to other and unspecified causes	G03	0-74	•	
**Neoplasms**				
Lip, oral cavity and pharynx cancer	C00-C14	0-74		•
Oesophageal cancer	C15	0-74		•
Stomach cancer	C16	0-74		•
Liver cancer	C22	0-74		•
Lung cancer	C33-C34	0-74		•
Mesothelioma	C45	0-74		•
Skin (melanoma) cancer	C43	0-74		•

Bladder cancer	C67	0-74		•
Cervical cancer	C53	0-74	• (50%)	• (50%)
Colorectal cancer	C18-C21	0-74	•	
Breast cancer (female only)	C50	0-74	•	
Uterus cancer	C54, C55	0-74	•	
Testicular cancer	C62	0-74	•	
Thyroid cancer	C73	0-74	•	
Hodgkin's disease	C81	0-74	•	
Lymphoid leukaemia	C91.0, C91.1	0-74	•	
Benign neoplasm	D10-D36	0-74	•	
**Endocrine and metabolic diseases**				
Nutritional deficiency anaemia	D50-D53	0-74		•
Diabetes mellitus	E10-E14	0-74	• (50%)	• (50%)
Thyroid disorders	E00-E07	0-74	•	
Adrenal disorders	E24-E25 (excl. E24.4), E27	0-74	•	
**Diseases of the nervous system**				
Epilepsy	G40, G41	0-74	•	
**Diseases of the circulatory system**				
Aortic aneurysm	I71	0-74	• (50%)	• (50%)
Hypertensive diseases	I10-I13, I15	0-74	• (50%)	• (50%)
Ischaemic heart diseases	I20-I25	0-74	• (50%)	• (50%)
Cerebrovascular diseases	I60-I69	0-74	• (50%)	• (50%)
Other atherosclerosis	I70, I73.9	0-74	• (50%)	• (50%)
Rheumatic and other heart diseases	I00-I09	0-74	•	
Venous thromboembolism	I26, I80, I82.9	0-74	•	
**Diseases of the respiratory system**				
Influenza	J09-J11	0-74		•
Pneumonia due to streptococcus pneumonia or haemophilus influenza	J13-J14	0-74		•
Chronic lower respiratory diseases	J40-J44	0-74		•

Lung diseases due to external agents	J60-J64, J66-J70, J82, J92	0-74		•
Upper respiratory infections	J00-J06, J30-J39	0-74	•	
Pneumonia, not elsewhere classified or organism unspecified	J12, J15, J16-J18	0-74	•	
Acute lower respiratory infections	J20-J22	0-74	•	
Asthma and bronchiectasis	J45-J47	0-74	•	
Adult respiratory distress syndrome	J80	0-74	•	
Pulmonary oedema	J81	0-74	•	
Abscess of lung and mediastinum pyothorax	J85, J86	0-74	•	
Other pleural disorders	J90, J93, J94	0-74	•	
**Diseases of the digestive system**				
Gastric and duodenal ulcer	K25-K28	0-74	•	
Appendicitis	K35-K38	0-74	•	
Abdominal hernia	K40-K46	0-74	•	
Cholelithiasis and cholecystitis	K80-K81	0-74	•	
Other diseases of gallbladder or biliary tract	K82-K83	0-74	•	
Acute pancreatitis	K85.0, K85.1, K85.3, K85.8, K85.9	0-74	•	
Other diseases of pancreas	K86.1, K86.2, K86.3, K86.8, K86.9	0-74	•	
**Diseases of the genitourinary system**				
Nephritis and nephrosis	N00-N07	0-74	•	
Obstructive uropathy	N13, N20-N21, N35	0-74	•	
Renal failure	N17-N19	0-74	•	
Renal colic	N23	0-74	•	
Disorders resulting from renal tubular dysfunction	N25	0-74	•	
Unspecified contracted kidney, small kidney of unknown cause	N26-N27	0-74	•	
Inflammatory diseases of genitourinary system	N34.1, N70-N73, N75.0, N75.1, N76.4, N76.6	0-74	•	
Prostatic hyperplasia	N40	0-74	•	

**Pregnancy, childbirth and the perinatal period**				
Tetanus neonatorum	A33	0-74		•
Obstetrical tetanus	A34	0-74		•
Pregnancy, childbirth and the puerperium	O00-O99	0-74	•	
Certain conditions originating in the perinatal period	P00-P96	0-74	•	
**Congenital malformations**				
Certain congenital malformations (neural tube defects)	Q00, Q01, Q05	0-74		•
Congenital malformations of the circulatory system (heart defects)	Q20-Q28	0-74	•	
**Adverse effects of medical and surgical care**				
Drugs, medicaments and biological substances causing adverse effects in therapeutic use	Y40-Y59	0-74	•	
Misadventures to patients during surgical and medical care	Y60-Y69, Y83-Y84	0-74	•	
Medical devices associated with adverse incidents in diagnostic and therapeutic use	Y70–Y82	0-74	•	
**Injuries**				
Transport Accidents	V01-V99	0-74		•
Accidental Injuries	W00-X39, X46-X59	0-74		•
Intentional self-harm	X66-X84	0-74		•
Event of undetermined intent	Y16-Y34	0-74		•
Assault	X86-Y09, U50.9	0-74		•
**Alcohol-related and drug-related deaths**				
Alcohol-specific disorders and poisonings	E24.4, F10, G31.2, G62.1, G72.1, I42.6, K29.2, K70, K85.2, K86.0, Q86.0, R78.0, X45, X65, Y15	0-74		•
Other alcohol-related disorders	K73, K74.0-K74.2, K74.6	0-74		•
Drug disorders and poisonings	F11-F16, F18-F19, X40-X44, X85, Y10-Y14	0-74		•
Intentional self-poisoning by drugs	X60-X64	0-74		•
**Provisional assignment of new diseases**				
COVID-19	U07.1-U07.2	0-74		•

A.7 – Data descriptives

A.7.1 – summary table

Below we present a summary table of the key variables of this paper.

Study Variables

	Min/ Max	Mean (SD)	Median (IQR)	Source
For-Profit Outsourcing (%)	-207.1/ 50.97	5.89 (0.48)	4.47 (22.3)	ONS
CCG Allocation (£000s per capita)	1.03/ 1.91	1.35 (0.01)	1.33 (3.54)	Rahal & Mohan, (2022).
LA Allocation (£000s per capita)	0.14/ 4.52	0.35 (0.01)	0.32 (0.2)	NHS England
Benefit Expenditure (£000s per capita)	0.06/ 2.42	1.12 (0.02)	1.03 (0.17)	MHCLG
Treatable Mortality Rate	49.1/ 187.1	85.52 (0.73)	82.75 (0.53)	DWP
CCG account balance (£000s)	-101774/ 5562	-20782.96 (605.75)	-17652 (16002.75)	NHS England
Treatment outsourcing (%)	0.16/ 51.49	8.86 (0.37)	6.43 (7.59)	NHS England

A.7.2 – summary by CCG

Study Variables (means)

CCG_Name	For-Profit Outsourcing (%)	CCG Allocation (£000s per capita)	LA Allocation (£000s per capita)	Benefit Expenditure (£000s per capita)	Treatable Mortality Rate	CCG account balance (£000s)	Treatment outsourcing (%)
AIREDALE WHARFEDALE AND CRAVEN	2.0040399	1.3200551	0.3927374	1.2339395	76.31111	-7758.667	10.8964534
ASHFORD	4.5548064	1.1813933	0.2822377	1.1045187	76.78889	-11187.571	8.6949541
AYLESBURY VALE	NaN	NaN	NaN	NaN	NaN	-12610.000	2.8900532
BARKING AND DAGENHAM	6.4209120	1.6057659	0.5496945	2.0363305	112.78000	-26619.167	16.9252487
BARNET	4.0750923	1.4935608	0.2772486	1.3814923	69.72222	-44292.714	0.8409992
BARNSLEY	2.5535948	1.7873400	0.4693370	1.6331018	98.92000	-23768.750	2.6038469
BASILDON AND BRENTWOOD	5.7858484	1.3381784	0.2756618	1.0012255	79.30000	-17200.000	16.3970335
BASSETLAW	5.0507408	1.5170380	0.3113625	1.2810198	88.23000	-8316.857	6.5152778
BATH AND NORTH EAST SOMERSET	12.7149831	1.4567778	0.2484815	0.8076593	69.32222	-13288.714	22.4126460
BATH AND NORTH EAST SOMERSET SWINDON AND WILTSHIRE	NaN	NaN	NaN	NaN	NaN	NaN	16.2643306
BEDFORDSHIRE	9.3148882	1.2068194	0.2588432	0.9805416	80.31000	-31862.875	2.8385431

Study Variables (means)

CCG_Name	For-Profit Outsourcing (%)	CCG Allocation (£000s per capita)	LA Allocation (£000s per capita)	Benefit Expenditure (£000s per capita)	Treatable Mortality Rate	CCG account balance (£000s)	Treatment outsourcing (%)
BEDFORDSHIRE LUTON AND MILTON KEYNES	NaN	NaN	NaN	NaN	NaN	NaN	4.8645702
BERKSHIRE WEST	NaN	1.2185410	0.2441986	0.8433799	78.20000	-34902.000	10.9715834
BEXLEY	2.9369950	1.2241388	0.2969126	1.1812782	77.58889	-26166.286	0.8399753
BIRMINGHAM AND SOLIHULL	5.1861274	1.4248881	0.4684026	1.4781800	101.45000	NaN	4.8924398
BIRMINGHAM CROSSCITY	NaN	0.7296326	NaN	NaN	NaN	-46050.250	3.2951096
BIRMINGHAM SOUTH AND CENTRAL	NaN	1.7519687	NaN	NaN	NaN	-24496.500	3.1481545
BLACK COUNTRY AND WEST BIRMINGHAM	NaN	NaN	NaN	NaN	NaN	NaN	5.0919595
BLACKBURN WITH DARWEN	6.9805565	1.5404663	0.5801595	1.9096274	116.54000	-7924.571	9.9511888
BLACKPOOL	8.5572793	1.9656360	0.6316682	2.2225272	139.71000	-11818.125	9.0735318
BOLTON	6.8668168	1.6150880	0.4892536	1.6559895	106.74000	-24418.125	10.3138171
BRACKNELL AND ASCOT	NaN	NaN	NaN	NaN	NaN	-13649.250	4.5776335
BRADFORD CITY	2.6976899	1.6149020	0.5209969	1.7027269	162.34444	-7362.167	22.8419312
BRADFORD DISTRICT AND CRAVEN	NaN	NaN	NaN	NaN	NaN	NaN	19.2497906
BRADFORD DISTRICTS	2.4629029	1.3184678	0.5209969	1.7027269	108.77778	-21629.000	23.5760208
BRENT	4.7993359	1.6285920	0.5503280	2.0354431	87.90000	-39706.000	4.9449167
BRIGHTON AND HOVE	6.3062925	1.5930929	0.4075509	1.4407780	84.32000	-20866.167	18.5665017
BRISTOL	NaN	NaN	NaN	NaN	NaN	-28132.000	9.9488374
BRISTOL NORTH SOMERSET AND SOUTH GLOUCESTERSHIRE	18.3517775	1.2618035	0.3221669	1.0945881	80.18000	-72822.000	13.8183661
BROMLEY	10.4205782	1.5155126	0.2200550	1.0274752	68.13333	-18846.429	9.3338067
BUCKINGHAMSHIRE	1.9777907	NaN	0.2161908	0.6470110	67.36000	-47314.000	3.4117330
BUCKINGHAMSHIRE OXFORDSHIRE AND BERKSHIRE WEST	NaN	NaN	NaN	NaN	NaN	NaN	NaN
BURY	4.3771741	1.5390974	0.3822926	1.4191020	97.90000	-15346.000	4.3104617
CALDERDALE	NaN	1.5679519	0.4023504	1.3729626	94.59000	-20635.875	12.0799573
CAMBRIDGESHIRE AND PETERBOROUGH	4.0284231	1.1627447	0.2828916	0.9653371	76.09000	-71829.500	5.2322138

Study Variables (means)

CCG_Name	For-Profit Outsourcing (%)	CCG Allocation (£000s per capita)	LA Allocation (£000s per capita)	Benefit Expenditure (£000s per capita)	Treatable Mortality Rate	CCG account balance (£000s)	Treatment outsourcing (%)
CAMDEN	24.7089249	1.9860996	0.7549636	1.6458007	72.56667	-47476.714	0.3234840
CANNOCK CHASE	1.3862433	1.2677137	0.2748545	1.0186111	82.41000	-8362.500	5.4509160
CANTERBURY AND COASTAL	5.1871529	1.2813067	0.2822377	1.2538242	75.32222	-19440.429	5.2730265
CASTLE POINT AND ROCHFORD	4.7488183	1.3357592	0.2756618	0.8982547	75.12000	-18324.125	5.5702944
CENTRAL LONDON WESTMINSTER	NaN	1.9429985	0.7999350	1.7438892	67.91000	-27977.500	0.4105991
CENTRAL MANCHESTER	NaN	NaN	NaN	NaN	NaN	-19986.500	5.5998732
CHESHIRE	NaN	NaN	NaN	NaN	NaN	NaN	7.3408989
CHILTERN	NaN	NaN	NaN	NaN	NaN	-24263.750	2.6744505
CHORLEY AND SOUTH RIBBLE	3.4192662	1.3977463	0.3706104	1.0552713	87.26000	-11029.500	14.8195281
CITY AND HACKNEY	3.4955461	1.8176686	5.5530912	1.6818652	108.31000	-54918.000	1.1184750
COASTAL WEST SUSSEX	3.1345752	1.3839791	0.2125597	0.9920836	76.95556	-38576.429	9.3157155
CORBY	2.6846243	1.3605871	0.3113381	1.3385046	111.00000	-5338.250	13.1585977
COUNTY DURHAM	NaN	NaN	NaN	NaN	NaN	NaN	5.0545231
COVENTRY AND RUGBY	3.0177230	1.3616422	0.3698993	1.2002836	93.57000	-42094.143	2.8250378
COVENTRY AND WARWICKSHIRE	NaN	NaN	NaN	NaN	NaN	NaN	5.3988440
CRAWLEY	1.3128621	1.4586997	0.2125597	1.2844166	91.84444	-3550.857	23.0340814
CROYDON	-38.8832847	1.4686577	0.3551956	1.5086876	90.81111	-37776.143	7.5130076
CUMBRIA	NaN	NaN	NaN	NaN	NaN	-23285.750	1.5396414
DARLINGTON	6.3804543	1.6277385	0.3988106	1.4534928	94.48889	-8128.286	14.3286522
DARTFORD GRAVESHAM AND SWANLEY	NaN	1.2203778	0.2822377	1.0363521	87.92222	-18981.333	4.4104954
DERBY AND DERBYSHIRE	3.7196120	1.4098329	0.3440149	1.2067130	86.71000	-63037.667	5.6533801
DEVON	3.5814419	NaN	0.3339668	1.1766369	77.66000	NaN	5.3498438
DONCASTER	8.8547291	1.4979440	0.4939511	1.5371216	99.46000	-31438.625	2.8690864
DORSET	4.2184648	1.3746890	0.2327566	0.2355753	72.35000	-48247.750	3.8955158
DUDLEY	4.2967370	1.3336466	0.4193858	1.3247973	89.90000	-25253.000	8.7298773
DURHAM DALES EASINGTON	6.3312577	1.6721165	0.4677314	1.5626017	100.42222	-26931.857	3.5981941

Study Variables (means)

CCG_Name	For-Profit Outsourcing (%)	CCG Allocation (£000s per capita)	LA Allocation (£000s per capita)	Benefit Expenditure (£000s per capita)	Treatable Mortality Rate	CCG account balance (£000s)	Treatment outsourcing (%)
AND SEDGEFIELD							
EALING	2.6683142	1.6662295	0.4453522	1.6642268	89.35000	-46220.800	2.0196174
EAST AND NORTH HERTFORDSHIRE	4.2560243	1.2722710	0.2412951	0.9185673	77.54000	-39161.143	7.8852932
EAST BERKSHIRE	1.8600623	1.2319564	0.2657702	0.9248234	79.76000	-63707.000	2.8827053
EAST LANCASHIRE	5.5337574	1.4704674	0.3706104	1.4677333	104.24000	-36357.429	7.0187645
EAST LEICESTERSHIRE AND RUTLAND	6.4004338	1.1199526	0.2245890	0.7611640	70.19000	-15598.500	5.9977567
EAST RIDING OF YORKSHIRE	6.8491964	1.2561370	0.3251442	0.9670587	81.45000	-26973.875	5.8722704
EAST STAFFORDSHIRE	16.1733558	1.2716390	0.2748545	1.0014964	89.54000	-9286.500	2.8390079
EAST SURREY	9.6730021	1.1690071	0.2005426	0.7657867	75.00000	-10668.167	15.6232433
EAST SUSSEX	NaN	NaN	NaN	NaN	NaN	NaN	16.6822197
EASTBOURNE HAILSHAM AND SEAFORD	2.4595383	1.4177578	0.2960271	1.0997955	77.70000	-17765.429	19.7192434
EASTERN CHESHIRE	39.8058678	1.2846323	0.1789344	0.8648080	69.91111	-14528.714	13.9535108
ENFIELD	4.3006309	1.4236114	0.4261441	1.9747890	79.47778	-37096.000	3.6144218
EREWASH	NaN	1.3379959	NaN	NaN	NaN	-9972.750	19.2005427
FAREHAM AND GOSPORT	3.2522821	1.2037573	0.1987067	0.9513756	75.72000	-13010.125	12.2726802
FRIMLEY	NaN	NaN	NaN	NaN	NaN	NaN	4.6990650
FYLDE AND WYRE	8.5572793	1.2115016	0.3706104	1.2643442	92.74000	-9320.625	10.0853375
GATESHEAD	NaN	1.8781130	NaN	NaN	NaN	NaN	NaN
GLOUCESTERSHIRE	2.7865148	1.2205178	0.2828771	0.9498518	76.57000	-42122.750	19.8720321
GREAT YARMOUTH AND WAVENEY	11.6217126	1.5417882	0.3577687	1.2250597	89.17778	-15106.833	0.4808054
GREATER HUDDERSFIELD	12.2885403	1.2540580	0.3665833	1.3797187	90.50000	-17473.000	21.4586644
GREATER MANCHESTER	NaN	NaN	NaN	NaN	NaN	NaN	NaN
GREATER PRESTON	3.8865152	1.3669466	0.3706104	1.0791275	98.31000	-13153.000	13.5077197
GREENWICH	10.4725214	1.6697212	0.6440112	1.6547790	98.43333	-26074.857	1.6115775
GUILDFORD AND WAVERLEY	NaN	1.2222740	0.2005426	0.6700853	65.11111	-17704.333	6.4154152
HALTON	1.6365939	1.5807160	0.5647861	1.7359998	107.44000	-6997.000	3.5624263

Study Variables (means)

CCG_Name	For-Profit Outsourcing (%)	CCG Allocation (£000s per capita)	LA Allocation (£000s per capita)	Benefit Expenditure (£000s per capita)	Treatable Mortality Rate	CCG account balance (£000s)	Treatment outsourcing (%)
HAMBLETON RICHMONDSHIRE AND WHITBY	1.4958993	1.2601966	0.2644779	0.9656807	71.80000	-11059.571	4.2722867
HAMMERSMITH AND FULHAM	1.9982777	1.7903590	0.6668926	1.5550319	89.57000	-25040.000	10.1026640
HAMPSHIRE SOUTHAMPTON AND ISLE OF WIGHT	NaN	NaN	NaN	NaN	NaN	NaN	8.6206895
HARDWICK	NaN	1.3400918	NaN	NaN	NaN	-7497.500	4.7750927
HARINGEY	20.2214363	1.6726038	0.5930048	2.0840628	94.72222	-34360.000	2.0197867
HARROGATE AND RURAL DISTRICT	0.3434487	1.2399156	0.2644779	0.7201543	71.27778	-10654.667	2.3214955
HARROW	6.3047602	1.3370466	0.2970035	1.3010840	73.53000	-29955.250	3.0302778
HARTLEPOOL AND STOCKTON ON TEES	2.4534226	1.6201875	0.4822827	1.6646762	99.31111	-21392.286	6.0295829
HASTINGS AND ROTHER	4.6265004	1.5096628	0.2960271	1.4498597	94.57778	-21214.000	5.1825609
HAVERING	NaN	1.6023419	0.2508444	1.1186116	81.24000	-31660.500	21.8074513
HEREFORDSHIRE	2.9363600	1.4183106	0.3208864	1.0132969	79.21111	-10755.571	6.2561405
HEREFORDSHIRE AND WORCESTERSHIRE	NaN	NaN	NaN	NaN	NaN	NaN	6.3983375
HERTS VALLEYS	5.3596605	1.2835255	0.2412951	0.8881219	75.04000	-54222.625	13.6978412
HEYWOOD MIDDLETON AND ROCHDALE	4.4850589	1.4646705	0.5736835	1.8047134	113.29000	-17838.500	14.4022761
HIGH WEALD LEWES HAVENS	13.6855562	1.2118629	0.2960271	0.9269461	65.24444	-8452.429	16.4802146
HILLINGDON	5.7233446	1.3943653	0.3200765	1.3148703	82.36000	-25786.250	1.8107334
HORSHAM AND MID SUSSEX	6.1212688	1.1426544	0.2125597	0.6961755	65.41111	-5631.600	27.0670803
HOUNSLOW	NaN	1.4946975	0.3795719	1.3505238	85.57000	-31304.750	0.1523533
HULL	17.9923413	1.5596903	0.6173006	1.9278734	119.78000	-23354.500	7.5200144
IPSWICH AND EAST SUFFOLK	7.5214499	1.1668289	0.3248573	0.8357226	73.59000	-30247.125	5.6843120
ISLE OF WIGHT	2.1391667	1.5531017	0.4556403	1.3951807	80.80000	-10902.875	2.6724900
ISLINGTON	NaN	1.9918083	0.7671900	1.9641141	98.84444	-31033.833	1.2596978
KENT AND MEDWAY	NaN	NaN	NaN	NaN	NaN	NaN	14.0631624
KERNOW	6.5310656	1.3797361	0.8338947	0.7952097	78.48000	-40748.875	8.0190262
KINGSTON	15.6041414	1.5597308	0.2367384	0.9419485	70.61111	-19093.857	0.8452703

Study Variables (means)

CCG_Name	For-Profit Outsourcing (%)	CCG Allocation (£000s per capita)	LA Allocation (£000s per capita)	Benefit Expenditure (£000s per capita)	Treatable Mortality Rate	CCG account balance (£000s)	Treatment outsourcing (%)
KIRKLEES	NaN	NaN	NaN	NaN	NaN	NaN	19.8859411
KNOWSLEY	4.1714680	2.0682619	0.8539420	2.2685157	114.54000	-8815.750	4.4187366
LAMBETH	1.2768142	1.8755141	0.7150545	1.7522522	101.13333	-28162.571	0.7140476
LANCASHIRE NORTH	NaN	NaN	NaN	NaN	NaN	-11104.750	5.0308581
LEEDS	4.5152885	1.4174116	0.4123428	1.3593460	95.63000	-56663.000	8.4842289
LEEDS NORTH	NaN	NaN	NaN	NaN	NaN	-12505.000	6.0390071
LEEDS SOUTH AND EAST	NaN	NaN	NaN	NaN	NaN	-14498.250	6.7949591
LEEDS WEST	NaN	NaN	NaN	NaN	NaN	-15158.500	8.6843647
LEICESTER CITY	5.5254972	1.2751643	0.5777424	1.7139688	111.80000	-24620.000	4.3809269
LEWISHAM	NaN	1.7589381	0.6520676	1.8556667	101.31111	-23899.167	0.8105600
LINCOLNSHIRE	NaN	NaN	NaN	NaN	NaN	NaN	8.9504107
LINCOLNSHIRE EAST	2.9514039	1.4861545	0.3720180	1.2494611	98.77778	-20226.571	8.2895793
LINCOLNSHIRE WEST	4.7107105	1.2262465	0.3720180	1.1668057	90.04444	-17306.857	9.0464460
LIVERPOOL	4.2740782	1.6580275	0.7280092	2.1203049	115.86000	-40904.250	7.7182482
LUTON	NaN	1.4332496	0.4447692	1.5827417	109.46000	-16893.571	2.0319534
MANCHESTER	NaN	NaN	0.7154594	1.9340289	137.96000	-56173.400	6.4644092
MANSFIELD AND ASHFIELD	5.5921191	1.3254886	0.3113625	1.5470516	101.12222	-12587.714	5.0825211
MEDWAY	17.6645506	1.4908065	0.3281881	1.3553763	94.35556	-30959.571	16.6874959
MERTON	2.7012556	1.4375216	0.3411342	1.1177074	79.52222	-14229.000	5.4227269
MID ESSEX	11.5616988	1.1706158	0.2756618	0.9199119	71.54000	-28726.500	18.8073220
MILTON KEYNES	4.6755496	1.2757416	0.3480829	1.0333659	86.77000	-21402.571	7.5633682
MORECAMBE BAY	3.2708439	NaN	0.3708268	1.1244631	85.32000	-22416.200	4.5750902
NENE	6.0572732	1.1940329	0.3113381	0.9174096	88.58889	-51277.143	9.8483388
NEWARK AND SHERWOOD	4.7264142	1.4084722	0.3113625	1.1440049	81.56667	-7715.286	6.9337615
NEWBURY AND DISTRICT	NaN	NaN	NaN	NaN	NaN	-10152.750	7.8270468
NEWCASTLE GATESHEAD	2.6461214	1.4939762	0.5789642	1.4503413	100.10000	-42237.714	2.6355701
NEWCASTLE NORTH AND EAST	NaN	1.8191773	NaN	NaN	NaN	-10987.500	NaN

Study Variables (means)

CCG_Name	For-Profit Outsourcing (%)	CCG Allocation (£000s per capita)	LA Allocation (£000s per capita)	Benefit Expenditure (£000s per capita)	Treatable Mortality Rate	CCG account balance (£000s)	Treatment outsourcing (%)
NEWCASTLE WEST	NaN	1.7819163	NaN	NaN	NaN	-11632.500	NaN
NEWHAM	0.2986233	1.6619755	0.6925016	2.0944409	106.68000	-52135.750	9.7684282
NORFOLK AND WAVENEY	NaN	NaN	NaN	NaN	NaN	NaN	3.1415044
NORTH AND WEST READING	NaN	NaN	NaN	NaN	NaN	-8442.750	12.8306595
NORTH CENTRAL LONDON	NaN	NaN	NaN	NaN	NaN	NaN	2.4341224
NORTH CUMBRIA	7.1318466	NaN	0.4063486	1.1252035	86.50000	-11693.500	1.9619496
NORTH DERBYSHIRE	NaN	1.4973029	NaN	NaN	NaN	-19552.000	7.9230178
NORTH DURHAM	4.0079823	1.4248383	0.4677314	1.5626017	86.24444	-20860.571	3.9299796
NORTH EAST ESSEX	12.3729726	1.3990203	0.2756618	1.3078142	86.28000	-26736.875	7.5609213
NORTH EAST HAMPSHIRE AND FARNHAM	6.2806245	1.2640041	0.1993187	0.7390661	67.22000	-16322.143	3.7801064
NORTH EAST LINCOLNSHIRE	19.2158001	1.4670463	0.4717101	1.4564448	97.81000	-9833.250	15.9103920
NORTH EAST LONDON	NaN	NaN	NaN	NaN	NaN	NaN	9.7542292
NORTH EAST WEST DEVON	NaN	0.4838870	NaN	NaN	NaN	NaN	NaN
NORTH EASTERN AND WESTERN DEVON	NaN	NaN	NaN	NaN	NaN	-45051.750	NaN
NORTH HAMPSHIRE	5.1801882	1.1346185	0.1987067	0.7007398	70.17000	-13447.500	3.1703851
NORTH KIRKLEES	12.2047592	1.3030189	0.3665833	1.3797187	97.16000	-14433.143	16.2036137
NORTH LINCOLNSHIRE	3.5370613	1.4767895	0.3906370	1.2322580	92.49000	-13366.500	12.1689126
NORTH MANCHESTER	NaN	NaN	NaN	NaN	NaN	-16899.750	8.9720212
NORTH NORFOLK	3.8102703	1.3774606	0.3906802	0.9339450	72.42222	-13959.714	1.3861487
NORTH SOMERSET	NaN	1.4512190	NaN	NaN	NaN	-13862.500	18.7453017
NORTH STAFFORDSHIRE	5.7029063	1.3282064	0.2748545	1.0264838	85.09000	-11969.167	7.2849798
NORTH TYNESIDE	1.6554586	1.7525009	0.4571006	1.3960881	91.98000	-18273.250	2.8975173
NORTH WEST LONDON	NaN	NaN	NaN	NaN	NaN	NaN	2.9925104
NORTH WEST SURREY	14.1994079	1.2665789	0.2005426	0.7598587	73.95556	-32316.429	2.4727107
NORTH YORKSHIRE	NaN	NaN	NaN	NaN	NaN	NaN	4.6988397
NORTHAMPTONSHIRE	NaN	NaN	NaN	NaN	NaN	NaN	9.2403544

Study Variables (means)

CCG_Name	For-Profit Outsourcing (%)	CCG Allocation (£000s per capita)	LA Allocation (£000s per capita)	Benefit Expenditure (£000s per capita)	Treatable Mortality Rate	CCG account balance (£000s)	Treatment outsourcing (%)
NORTHERN EASTERN AND WESTERN DEVON	NaN	1.0789355	NaN	NaN	NaN	NaN	4.7324063
NORTHUMBERLAND	NaN	1.6157039	0.4229489	1.0567500	82.66000	-15559.250	1.2856719
NORWICH	3.6755734	1.1547750	0.3906802	1.0717667	81.55556	-18597.667	2.0772496
NOTTINGHAM AND NOTTINGHAMSHIRE	NaN	NaN	NaN	NaN	NaN	NaN	13.5298690
NOTTINGHAM CITY	19.4359531	1.6259567	0.5815084	1.8324114	118.62222	-24587.714	30.0549802
NOTTINGHAM NORTH AND EAST	8.3949923	1.2463496	0.3113625	1.2545154	83.24444	-6600.000	26.8606889
NOTTINGHAM WEST	8.8560264	1.0716082	0.3113625	1.0041340	80.22222	-4147.429	30.5987469
OLDHAM	8.2884770	1.6825331	0.5658750	1.7146042	110.15000	-31949.500	40.9783128
OXFORDSHIRE	2.6791276	1.1186089	0.2442488	0.7942622	71.67000	-42296.625	7.3807304
PORTSMOUTH	4.6880558	1.5018638	0.4491347	1.4356795	96.12000	-12172.667	14.4069519
REDBRIDGE	NaN	1.1551090	0.3514613	1.3923040	85.31000	-29483.167	21.9583428
REDDITCH AND BROMSGROVE	2.8556868	1.1299817	0.2460617	1.0067499	82.53333	-5686.714	6.1175082
RICHMOND	2.9762075	1.4468640	0.1882854	0.7611258	65.77778	-22536.000	0.2221765
ROTHERHAM	3.1659587	1.6008452	0.4767774	1.6144837	101.71000	-20993.000	2.5458323
RUSHCLIFFE	11.9324556	1.2302479	0.3113625	0.6897779	69.98889	-11394.714	28.3246475
SALFORD	3.0672487	1.8256864	0.6128450	1.8555344	117.12000	-14143.500	8.8040284
SANDWELL AND WEST BIRMINGHAM	4.6345327	0.9681984	0.6381453	1.8595168	118.00000	-50603.429	1.7070940
SCARBOROUGH AND RYEDALE	5.3799749	1.4852452	0.2644779	1.1239637	95.12222	-11695.714	1.6205471
SHEFFIELD	3.6492285	1.3488746	0.5072482	1.4302704	88.16000	-34421.250	3.3126780
SHROPSHIRE	3.3368759	1.3882564	0.3117914	0.9517960	77.40000	-20763.500	3.0842533
SHROPSHIRE TELFORD AND WREKIN	NaN	NaN	NaN	NaN	NaN	NaN	7.2279130
SLOUGH	NaN	NaN	NaN	NaN	NaN	-13606.000	2.2798163
SOLIHULL	NaN	NaN	NaN	NaN	NaN	-18068.000	5.6648116
SOMERSET	4.4678616	1.3234996	0.2933782	0.9349749	70.66000	-35085.875	9.3460096
SOUTH CHESHIRE	3.7285734	1.2529310	0.1789344	0.8648080	89.01111	-12852.143	4.5714638

Study Variables (means)

CCG_Name	For-Profit Outsourcing (%)	CCG Allocation (£000s per capita)	LA Allocation (£000s per capita)	Benefit Expenditure (£000s per capita)	Treatable Mortality Rate	CCG account balance (£000s)	Treatment outsourcing (%)
SOUTH DEVON AND TORBAY	NaN	1.4776570	NaN	NaN	NaN	-15019.500	4.5054190
SOUTH EAST LONDON	NaN	NaN	NaN	NaN	NaN	NaN	2.4624521
SOUTH EAST STAFFORDSHIRE AND SEISDON AND PENINSULAR	NaN	NaN	NaN	NaN	NaN	-11877.250	NaN
SOUTH EAST STAFFORDSHIRE AND SEISDON PENINSULA	1.6878548	1.1405894	0.2748545	1.0037435	79.11000	-14916.500	2.5005263
SOUTH EAST STAFFS AND SEISDON AND PENINSULAR	NaN	NaN	NaN	NaN	NaN	NaN	NaN
SOUTH EASTERN HAMPSHIRE	4.2043080	1.2196531	0.1987067	0.8634490	71.10000	-16592.625	9.6103697
SOUTH GLOUCESTERSHIRE	NaN	1.2563881	NaN	NaN	NaN	-16425.000	13.0641684
SOUTH KENT COAST	7.5826348	1.3783420	0.2822377	1.2404517	87.67778	-21575.143	4.0123674
SOUTH LINCOLNSHIRE	3.3389988	1.3929495	0.3720180	1.0004585	80.81111	-12537.714	7.9530941
SOUTH MANCHESTER	NaN	NaN	NaN	NaN	NaN	-10718.500	6.2274482
SOUTH NORFOLK	4.3562387	1.1786005	0.3906802	0.9400623	69.47778	-15449.286	2.4047420
SOUTH READING	NaN	NaN	NaN	NaN	NaN	-7163.000	13.7820397
SOUTH SEFTON	2.9589145	1.5739342	0.4692111	1.5329889	99.75000	-13384.500	6.7862268
SOUTH TEES	2.7691997	1.2944538	0.5472237	1.9045852	111.68889	-21793.857	7.7415281
SOUTH TYNESIDE	2.3728609	1.8987494	0.6375589	1.7667364	96.71000	-16238.875	7.2529382
SOUTH WARWICKSHIRE	3.0821571	1.2570660	0.2632533	0.7809500	72.80000	-10254.286	3.1750378
SOUTH WEST LINCOLNSHIRE	3.5795178	1.3128957	0.3720180	0.9251260	83.93333	-10436.286	8.5359894
SOUTH WEST LONDON	NaN	NaN	NaN	NaN	NaN	NaN	2.4333793
SOUTH WORCESTERSHIRE	4.0169131	1.1569157	0.2460617	1.0094666	77.18889	-15045.571	8.0648921
SOUTHAMPTON	5.7321260	1.5517885	0.4439781	1.3692611	93.81000	-18536.250	31.1325310
SOUTHEND	4.8076836	1.3610279	0.3767200	1.4295623	89.65000	-16979.000	4.6069102
SOUTHERN DERBYSHIRE	NaN	1.2935321	NaN	NaN	NaN	-40380.500	2.9563906
SOUTHPORT AND FORMBY	4.3613847	1.6064625	0.4692111	1.5329889	81.40000	-10709.000	11.1957846
SOUTHWARK	1.2096786	1.6885021	0.8092886	1.6654548	96.77778	-24484.167	0.7253536
ST HELENS	NaN	1.6873106	0.5197867	1.7172501	102.33000	-10398.500	6.4073830
STAFFORD AND SURROUNDS	9.5635580	1.1450291	0.2748545	0.8665473	73.63000	-17125.833	7.2278226

Study Variables (means)

CCG_Name	For-Profit Outsourcing (%)	CCG Allocation (£000s per capita)	LA Allocation (£000s per capita)	Benefit Expenditure (£000s per capita)	Treatable Mortality Rate	CCG account balance (£000s)	Treatment outsourcing (%)
STOCKPORT	5.7010103	1.5552155	0.3262755	1.1817273	82.45000	-21238.375	5.6806639
STOKE ON TRENT	7.9147498	1.5172780	0.4157966	1.3381703	102.77000	-13210.750	6.8880441
SUNDERLAND	4.2972936	1.8659543	0.6079469	1.7802746	100.39000	-28485.750	2.6839759
SURREY DOWNS	14.7802062	1.2591072	0.2005426	0.7114741	62.45556	-41762.857	27.5841329
SURREY HEARTLANDS	NaN	NaN	NaN	NaN	NaN	NaN	11.4950703
SURREY HEATH	1.3044954	1.2550714	0.2005426	0.6561314	59.86000	-5427.571	13.8170941
SUTTON	2.9583098	1.4471390	0.3253991	1.0444585	74.27778	-16047.714	1.8005202
SWALE	NaN	1.2366729	0.2822377	1.4106882	91.77778	-15216.500	14.7358165
SWINDON	6.0622261	1.3416628	0.2591154	0.8871701	87.56667	-15076.000	9.4536982
TAMESIDE AND GLOSSOP	4.7441940	1.5475705	0.4171132	1.3624870	106.34000	-16646.500	9.0314062
TEES VALLEY	NaN	NaN	NaN	NaN	NaN	NaN	10.2348723
TELFORD AND WREKIN	1.3331075	1.4087909	0.4360138	1.5590270	97.68000	-13420.500	3.5832731
THANET	2.8019933	1.5346661	0.2822377	1.7180116	100.25556	-12693.286	3.8027651
THURROCK	2.6411420	1.2392838	0.3863557	1.2480661	88.88000	-12791.500	9.7594639
TOWER HAMLETS	NaN	1.6839869	0.8698695	2.0388443	101.05000	-54559.571	9.9339224
TRAFFORD	3.9934938	1.3096014	0.3308060	1.1056695	83.16000	-19693.500	5.1937053
VALE OF YORK	0.8981463	1.1549735	0.2728808	0.8409022	79.93000	-20127.125	4.4711398
VALE ROYAL	2.7766693	1.2730936	0.2631530	1.0884294	87.25556	-7573.857	4.7790273
WAKEFIELD	4.9741473	1.5143236	0.4327119	1.5465631	98.23000	-28791.250	19.5013083
WALSALL	3.1947509	1.5980008	0.5158921	1.6745361	102.85000	-26150.000	3.7575718
WALTHAM FOREST	4.6716208	1.2820689	0.5027260	1.7008526	95.26000	-23534.714	15.0473718
WANDSWORTH	NaN	1.6168296	0.4848656	1.2898362	83.94444	-29058.667	1.7493382
WARRINGTON	3.0191161	1.4825199	0.2806174	1.1282495	91.53000	-13529.286	6.3145675
WARWICKSHIRE NORTH	3.1881692	1.2307206	0.2632533	1.1846573	93.07000	-17169.714	2.8479504
WEST CHESHIRE	3.0431326	1.4761978	0.2631530	1.0884294	79.06667	-11689.000	3.6066711
WEST ESSEX	3.1990560	1.2608954	0.2756618	0.9844610	76.95000	-21520.000	10.2332536
WEST HAMPSHIRE	5.4708901	1.1922937	0.1987067	0.7837889	62.88000	-46347.375	4.6083264

Study Variables (means)

CCG_Name	For-Profit Outsourcing (%)	CCG Allocation (£000s per capita)	LA Allocation (£000s per capita)	Benefit Expenditure (£000s per capita)	Treatable Mortality Rate	CCG account balance (£000s)	Treatment outsourcing (%)
WEST KENT	3.7627633	1.1481021	0.2822377	0.8829893	73.90000	-28035.000	12.7781763
WEST LANCASHIRE	6.1785737	1.3591499	0.3706104	1.2401941	86.26000	-7297.750	12.9755151
WEST LEICESTERSHIRE	4.9134165	1.0874790	0.2273051	0.8123710	76.26000	-21038.571	4.2087393
WEST LONDON	39.0699237	1.8745667	0.7279895	1.6037220	65.51000	-61787.000	0.5263075
WEST LONDON KANDC AND QPP	NaN	NaN	NaN	NaN	NaN	-50062.750	NaN
WEST NORFOLK	4.2388663	1.3892893	0.3906802	1.0826297	81.18889	-13137.286	5.1114118
WEST SUFFOLK	6.4881571	1.3419430	0.3248573	0.6866124	67.59000	-18624.625	2.0192400
WEST SUSSEX	NaN	NaN	NaN	NaN	NaN	NaN	19.3060582
WIGAN BOROUGH	3.4951655	1.6352259	0.4517052	1.4433375	106.55000	-23696.750	5.3440831
WILTSHIRE	11.7715276	1.1954885	0.2222053	0.8545283	73.48889	-34137.000	17.7326253
WINDSOR ASCOT AND MAIDENHEAD	NaN	NaN	NaN	NaN	NaN	-10085.000	1.6584402
WIRRAL	4.4405010	1.5992971	0.5086710	1.7014497	94.26000	-26561.125	5.2572582
WOKINGHAM	NaN	NaN	NaN	NaN	NaN	-9948.000	11.5309470
WOLVERHAMPTON	NaN	1.6233011	0.6051321	1.7231589	114.96000	-27804.167	7.1281057
WYRE FOREST	4.1867039	1.4198590	0.2460617	1.2493154	85.87778	-7748.571	5.0785388

A.7.3 – summary table by year

Variable	N	Year						
		2014, N = 185	2015, N = 185	2016, N = 185	2017, N = 186	2018, N = 180	2019, N = 185	2020, N = 112
**For-Profit Outsourcing (%)**	782							
N		110	115	117	120	128	139	53
Mean (Median)		5.7 (3.2)	5.6 (3.8)	6.1 (4.0)	6.9 (4.8)	5.1 (4.6)	6.9 (5.1)	7.3 (5.2)
Range (SD)		0.4, 58.0 (7.6)	0.5, 51.0 (6.6)	-1.3, 49.9 (6.8)	0.0, 33.9 (6.3)	-207.1, 39.3 (19.9)	0.3, 51.2 (6.1)	1.9, 33.5 (5.8)
**CCG Allocation (£000s per capita)**	1,218							
N		185	185	185	186	180	185	112
Mean (Median)		1.30 (1.29)	1.31 (1.29)	1.33 (1.31)	1.37 (1.34)	1.36 (1.34)	1.36 (1.34)	1.41 (1.40)
Range (SD)		1.01, 1.82 (0.16)	1.03, 1.83 (0.16)	1.08, 1.84 (0.15)	1.11, 1.91 (0.15)	1.10, 1.92 (0.15)	1.09, 1.94 (0.15)	1.11, 2.14 (0.16)
**LA Allocation (£000s per capita)**	1,194							
N		179	179	179	180	180	185	112
Mean (Median)		0.52 (0.45)	0.47 (0.41)	0.39 (0.36)	0.35 (0.30)	0.32 (0.27)	0.31 (0.26)	0.31 (0.26)
Range (SD)		0.26, 5.31 (0.40)	0.24, 4.52 (0.34)	0.22, 1.32 (0.15)	0.16, 4.08 (0.30)	0.13, 3.36 (0.26)	0.13, 2.99 (0.24)	0.12, 3.28 (0.31)
**Benefit Expenditure (£000s per capita)**	1,192							

Variable	N	Year						
		2014, N = 185	2015, N = 185	2016, N = 185	2017, N = 186	2018, N = 180	2019, N = 185	2020, N = 112
N		179	179	179	180	180	183	112
Mean (Median)		1.40 (1.38)	1.37 (1.35)	1.31 (1.29)	1.20 (1.18)	0.83 (0.80)	0.90 (0.86)	0.61 (0.60)
Range (SD)		0.09, 2.51 (0.43)	0.07, 2.43 (0.42)	0.06, 2.29 (0.40)	0.07, 2.09 (0.38)	0.36, 1.50 (0.26)	0.42, 1.65 (0.28)	0.32, 1.11 (0.18)
**Treatable Mortality Rate**	1,008							
N		179	179	179	180	180	111	0
Mean (Median)		87 (85)	87 (84)	87 (86)	85 (82)	85 (83)	85 (83)	NA (NA)
Range (SD)		58, 129 (16)	59, 176 (17)	57, 187 (17)	45, 140 (17)	48, 147 (16)	47, 145 (16)	Inf, -Inf (NA)
**CCG account balance (£000s)**	1,137							
N		181	181	182	184	152	168	89
Mean (Median)		-17,248 (-15,386)	-18,373 (-15,592)	-19,864 (-17,657)	-19,844 (-17,162)	-23,449 (-20,905)	-26,419 (-22,801)	-31,689 (-27,210)
Range (SD)		-47,652, -403 (9,192)	-52,246, -3,611 (10,793)	-58,371, -876 (11,980)	-56,590, 5,562 (11,964)	-101,774, -2,751 (15,029)	-96,792, 3,357 (16,596)	-94,147, 6,964 (20,160)
**Treatment outsourcing (%)**	1,033							
N		0	185	185	186	180	185	112
Mean (Median)		NA (NA)	8 (5)	7 (5)	8 (6)	9 (6)	9 (6)	7 (5)
Range (SD)		Inf, -Inf (NA)	0, 49 (9)	0, 38 (8)	0, 44 (8)	0, 51 (9)	0, 43 (9)	0, 45 (6)

A.6 – Data visualisation

Figure 1 in the main paper presents the impacts of austerity on reduced NHS funding, here we show the same plots for LA and benefit funding/expenditure.

A.6.1 – LA Allocation

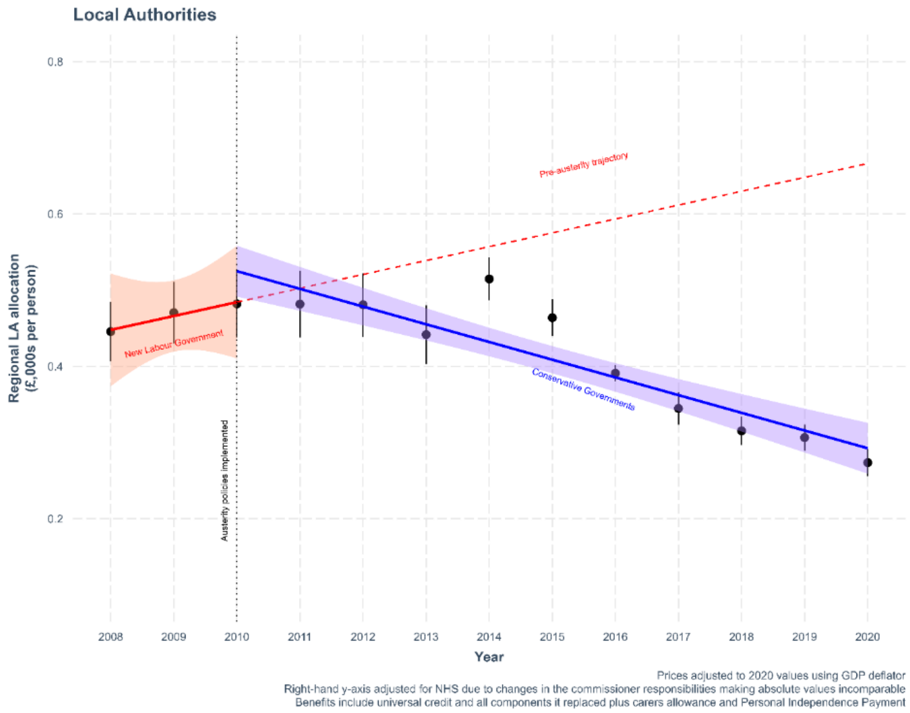

A.6.2 Benefits expenditure

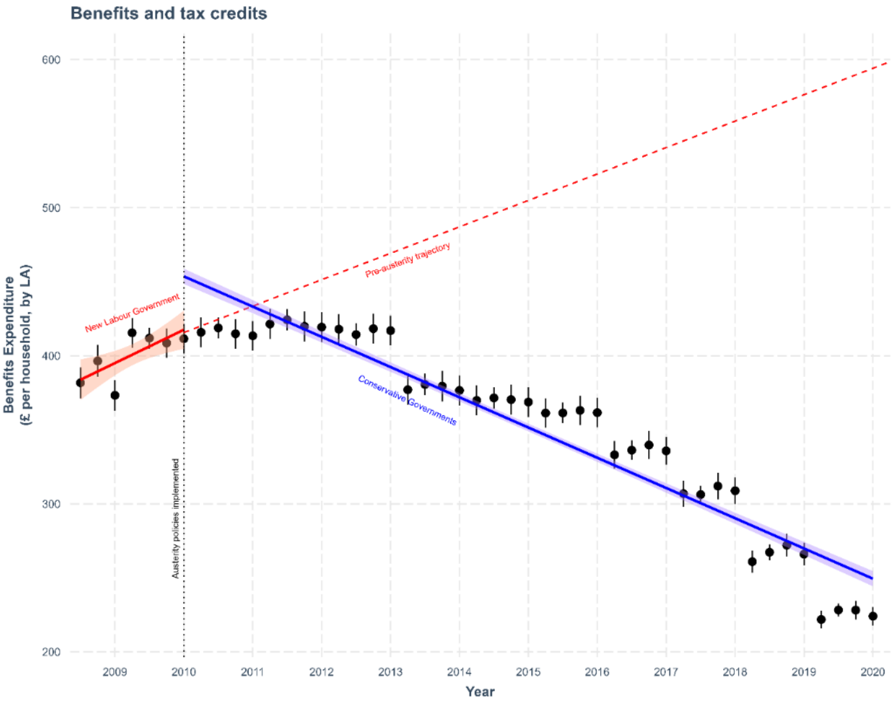

Supplementary Analyses

A.8 Confounding privatisation

	ln. Treatable Mortality [.95 ci]	p-value	ln. Treatable Mortality [.95 ci]	p-value	ln. Treatable Mortality [.95 ci]	p-value	ln. Treatable Mortality [.95 ci]	p-value	ln. Treatable Mortality [.95 ci]	p-value
For-profit outsourcing (%)	0.0039 [0.0020, 0.0059]	0.0043	0.0039 [0.0020, 0.0057]	0.0040	0.0040 [0.0020, 0.0060]	0.0039	0.0039 [0.0019, 0.0059]	0.0044	0.0039 [0.0020, 0.0058]	0.0038
CCG Allocation (£ per capita)			-0.1343 [-0.5457, 0.2772]	0.5264					-0.1290 [-0.5428, 0.2848]	0.5449
LA Allocation (£ per capita)					0.0507 [-0.0888, 0.1902]	0.4850			0.0252 [-0.1179, 0.1684]	0.7326
Benefit Allocation (£ per capita)							-0.0390 [-0.0848, 0.0068]	0.2960	-0.0405 [-0.0973, 0.0162]	0.3058
Total CCG Spend (£10ms)	-0.0008 [-0.0016, 0.0000]	0.0804	-0.0007 [-0.0016, 0.0002]	0.1668	-0.0008 [-0.0016, 0.0001]	0.0955	-0.0009 [-0.0017, -0.0001]	0.0544	-0.0008 [-0.0016, 0.0001]	0.1167
Average Income (£)	0.0000 [0.0000, 0.0000]	0.6210	0.0000 [0.0000, 0.0000]	0.6617	0.0000 [0.0000, 0.0000]	0.6867	0.0000 [0.0000, 0.0000]	0.6279	0.0000 [0.0000, 0.0000]	0.6938
CCG Population	0.0000 [0.0000, 0.0000]	0.4871	0.0000 [0.0000, 0.0000]	0.5644	0.0000 [0.0000, 0.0000]	0.4378	0.0000 [0.0000, 0.0000]	0.4572	0.0000 [0.0000, 0.0000]	0.5090
Num.Obs.	500	500	482	482	500	500	500	500	482	482
R2	0.050	0.050	0.048	0.048	0.052	0.052	0.054	0.054	0.053	0.053
R2 Adj.	-0.354	-0.354	-0.359	-0.359	-0.356	-0.356	-0.353	-0.353	-0.360	-0.360
AIC	4619.9	4619.9	4473.7	4473.7	4621.2	4621.2	4620.0	4620.0	4475.3	4475.3
BIC	4641.0	4641.0	4498.7	4498.7	4646.5	4646.5	4645.3	4645.3	4508.7	4508.7
RMSE	0.05	0.05	0.05	0.05	0.05	0.05	0.05	0.05	0.05	0.05
CCG Fixed Effects	Yes	Yes	Yes	Yes	Yes	Yes	Yes	Yes	Yes	Yes
Time Fixed Effects	Yes	Yes	Yes	Yes	Yes	Yes	Yes	Yes	Yes	Yes
Clustered Standard Errors	Yes	Yes	Yes	Yes	Yes	Yes	Yes	Yes	Yes	Yes
Control variables	Yes	Yes	Yes	Yes	Yes	Yes	Yes	Yes	Yes	Yes

Table reports results from multivariate longitudinal regression models.

Robust SEs are clustered at CCG level and use a bias-reduced linearization estimator (CR2)

Lag of one year applied to allocation variables

	ln. Treatable Mortality [.95 ci]	p-value	ln. Treatable Mortality [.95 ci]	p-value	ln. Treatable Mortality [.95 ci]	p-value	ln. Treatable Mortality [.95 ci]	p-value	ln. Treatable Mortality [.95 ci]	p-value

Tr. mortality and allocations are log transformed, "Ln" denotes the natural log of outcome variable.

Control variables are household income, total commissioner spend and population size

A.2 Moderating privatisation

	ln. Treatable Mortality [.95 ci]	p-value	ln. Treatable Mortality [.95 ci]	p-value	ln. Treatable Mortality [.95 ci]	p-value	ln. Treatable Mortality [.95 ci]	p-value
For-profit outsourcing (%)	0.0039 [0.0020, 0.0059]	0.0043	0.0057 [0.0021, 0.0093]	0.0122	0.0075 [0.0040, 0.0110]	0.0041	0.0041 [0.0024, 0.0057]	0.0011
CCG Allocation (£ per capita)			-0.0851 [-0.5257, 0.3556]	0.7074				
LA Allocation (£ per capita)					0.0276 [-0.1155, 0.1707]	0.7098		
Benefit Allocation (£ per capita)							-0.0567 [-0.0977, -0.0158]	0.1378
Interaction: Outsourcing*CCG_Allocation			-0.0078 [-0.0237, 0.0081]	0.3615				
Interaction: Outsourcing*LA_Allocation					0.0030 [-0.0001, 0.0061]	0.0902		
Interaction: Outsourcing*Bens_Allocation							0.0040 [0.0005, 0.0075]	0.0483
Num.Obs.	500	500	482	482	500	500	500	500
R2	0.050	0.050	0.049	0.049	0.057	0.057	0.062	0.062
R2 Adj.	-0.354	-0.354	-0.361	-0.361	-0.352	-0.352	-0.345	-0.345
AIC	4619.9	4619.9	4475.0	4475.0	4620.5	4620.5	4617.9	4617.9
BIC	4641.0	4641.0	4504.2	4504.2	4650.0	4650.0	4647.4	4647.4
RMSE	0.05	0.05	0.05	0.05	0.05	0.05	0.05	0.05
CCG Fixed Effects	Yes	Yes	Yes	Yes	Yes	Yes	Yes	Yes
Time Fixed Effects	Yes	Yes	Yes	Yes	Yes	Yes	Yes	Yes
Clustered Standard Errors	Yes	Yes	Yes	Yes	Yes	Yes	Yes	Yes
Control variables	Yes	Yes	Yes	Yes	Yes	Yes	Yes	Yes

Table reports results from multivariate longitudinal regression models.

Robust SEs are clustered at CCG level and use a bias-reduced linearization estimator (CR2)

Lag of one year applied to allocation variables

Tr. mortality and allocations are log transformed, "Ln" denotes the natural log of outcome variable.

Control variables are household income, total commissioner spend and population size

A.5 Confounding privatisation on avoidable mortality

	ln. Avoidable Mortality [.95 ci]	p-value	ln. Avoidable Mortality [.95 ci]	p-value	ln. Avoidable Mortality [.95 ci]	p-value	ln. Avoidable Mortality [.95 ci]	p-value	ln. Avoidable Mortality [.95 ci]	p-value
For-profit outsourcing (%)	0.0020 [0.0005, 0.0035]	0.0314	0.0020 [0.0005, 0.0035]	0.0309	0.0020 [0.0004, 0.0035]	0.0338	0.0020 [0.0005, 0.0035]	0.0314	0.0020 [0.0005, 0.0035]	0.0349
CCG Allocation (£ per capita)			-0.1878 [-0.5575, 0.1819]	0.3260					-0.1828 [-0.5538, 0.1881]	0.3404
LA Allocation (£ per capita)					-0.0048 [-0.1091, 0.0994]	0.9287			-0.0183 [-0.1237, 0.0872]	0.7376
Benefit Allocation (£ per capita)							-0.0331 [-0.0588, -0.0073]	0.1890	-0.0341 [-0.0576, -0.0106]	0.1143
Num.Obs.	500	500	482	482	500	500	500	500	482	482
R2	0.030	0.030	0.036	0.036	0.030	0.030	0.035	0.035	0.042	0.042
R2 Adj.	-0.383	-0.383	-0.375	-0.375	-0.387	-0.387	-0.380	-0.380	-0.376	-0.376
AIC	5695.9	5695.9	5505.6	5505.6	5697.9	5697.9	5695.3	5695.3	5507.0	5507.0
BIC	5717.0	5717.0	5530.7	5530.7	5723.2	5723.2	5720.6	5720.6	5540.4	5540.4
RMSE	0.04	0.04	0.04	0.04	0.04	0.04	0.04	0.04	0.04	0.04
CCG Fixed Effects	Yes	Yes	Yes	Yes	Yes	Yes	Yes	Yes	Yes	Yes
Time Fixed	Yes	Yes	Yes	Yes	Yes	Yes	Yes	Yes	Yes	Yes

	ln. Avoidable Mortality [.95 ci]	p-value	ln. Avoidable Mortality [.95 ci]	p-value	ln. Avoidable Mortality [.95 ci]	p-value	ln. Avoidable Mortality [.95 ci]	p-value	ln. Avoidable Mortality [.95 ci]	p-value
Effects										
Clustered Standard Errors	Yes	Yes	Yes	Yes	Yes	Yes	Yes	Yes	Yes	Yes
Control variables	Yes	Yes	Yes	Yes	Yes	Yes	Yes	Yes	Yes	Yes

Table reports results from multivariate longitudinal regression models.

Robust SEs are clustered at CCG level and use a bias-reduced linearization estimator (CR2)

Lag of one year applied to allocation variables

Tr. mortality and allocations are log transformed, "Ln" denotes the natural log of outcome variable.

Control variables are household income, total commissioner spend and population size

A.3 CCG Balance

	Relationship		Confounding		Moderating	
	For-profit Outsourcing (%) [.95 ci]	p-value	ln. Treatable Mortality [.95 ci]	p-value	ln. Treatable Mortality [.95 ci]	p-value
For-profit outsourcing (%)			0.0044 [0.0023, 0.0064]	0.0016	0.0047 [0.0018, 0.0075]	0.0122
CCG Balance (£000s)	0.0000 [-0.0001, 0.0001]	0.5237	0.0000 [0.0000, 0.0000]	0.7144	0.0000 [0.0000, 0.0000]	0.6692
Interaction: Outsourcing*Balance					0.0000 [0.0000, 0.0000]	0.7380
Num.Obs.	457	457	457	457	457	457
R2	0.002	0.002	0.045	0.045	0.045	0.045
R2 Adj.	-0.440	-0.440	-0.396	-0.396	-0.401	-0.401
AIC	2138.1	2138.1	4386.9	4386.9	4388.9	4388.9
BIC	2146.4	2146.4	4411.7	4411.7	4417.7	4417.7
RMSE	2.50	2.50	0.05	0.05	0.05	0.05
CCG Fixed Effects	Yes	Yes	Yes	Yes	Yes	Yes
Time Fixed Effects	Yes	Yes	Yes	Yes	Yes	Yes
Clustered Standard Errors	Yes	Yes	Yes	Yes	Yes	Yes
Control variables	No	No	Yes	Yes	Yes	Yes

Table reports results from multivariate longitudinal regression models.

Robust SEs are clustered at CCG level and use a bias-reduced linearization estimator (CR2)

Lag of one year applied to independent variables

Tr. mortality are log transformed, "Ln" denotes the natural log of outcome variable.

Control variables are household income, total commissioner spend and population size

A.4 Private sector treatments

	**Treatments Outsourced (%) [.95 ci]**	**p-value**	**Treatments Outsourced (%) [.95 ci]**	**p-value**	**Treatments Outsourced (%) [.95 ci]**	**p-value**	**ln. Treatable Mortality [.95 ci]**	**p-value**	**ln. Treatable Mortality [.95 ci]**	**p-value**	**ln. Treatable Mortality [.95 ci]**	**p-value**
Treatments outsourced (%)							0.0018 [-0.0002, 0.0039]	0.0977	0.0019 [-0.0002, 0.0040]	0.1061	0.0018 [-0.0003, 0.0040]	0.1121
CCG Allocation (£ per capita)	9.9927 [-35.9020, 55.8874]	0.6720					-0.0643 [-0.5519, 0.4233]	0.7969				
LA Allocation (£ per capita)			0.6469 [-2.6461, 3.9398]	0.7375					-0.0073 [-0.0797, 0.0651]	0.8551		
Benefit Allocation (£ per capita)					0.0314 [-4.9454, 5.0081]	0.9917					-0.0339 [-0.0972, 0.0295]	0.4181
Num.Obs.	438	438	448	448	448	448	644	644	660	660	660	660
R2	0.002	0.002	0.001	0.001	0.000	0.000	0.012	0.012	0.012	0.012	0.014	0.014
R2 Adj.	-0.569	-0.569	-0.590	-0.590	-0.590	-0.590	-0.393	-0.393	-0.406	-0.406	-0.403	-0.403
AIC	2320.1	2320.1	2363.3	2363.3	2363.4	2363.4	5606.2	5606.2	5748.8	5748.8	5747.7	5747.7
BIC	2336.4	2336.4	2379.8	2379.8	2379.9	2379.9	5633.0	5633.0	5775.8	5775.8	5774.6	5774.6
RMSE	3.39	3.39	3.35	3.35	3.35	3.35	0.06	0.06	0.06	0.06	0.06	0.06
CCG Fixed Effects	Yes	Yes	Yes	Yes	Yes	Yes	Yes	Yes	Yes	Yes	Yes	Yes
Time Fixed Effects	Yes	Yes	Yes	Yes	Yes	Yes	Yes	Yes	Yes	Yes	Yes	Yes
Clustered Standard Errors	Yes	Yes	Yes	Yes	Yes	Yes	Yes	Yes	Yes	Yes	Yes	Yes
Control variables	Yes	Yes	Yes	Yes	Yes	Yes	Yes	Yes	Yes	Yes	Yes	Yes

Table reports results from multivariate longitudinal regression models.

Robust SEs are clustered at CCG level and use a bias-reduced linearization estimator (CR2)

Lag of one year applied to allocation variables

Tr. mortality and allocations are log transformed, "Ln" denotes the natural log of outcome variable.

Control variables are household income, total commissioner spend and population size

A.9. Data breakdowns

A.9.1 Breakdowns of data by treatment type

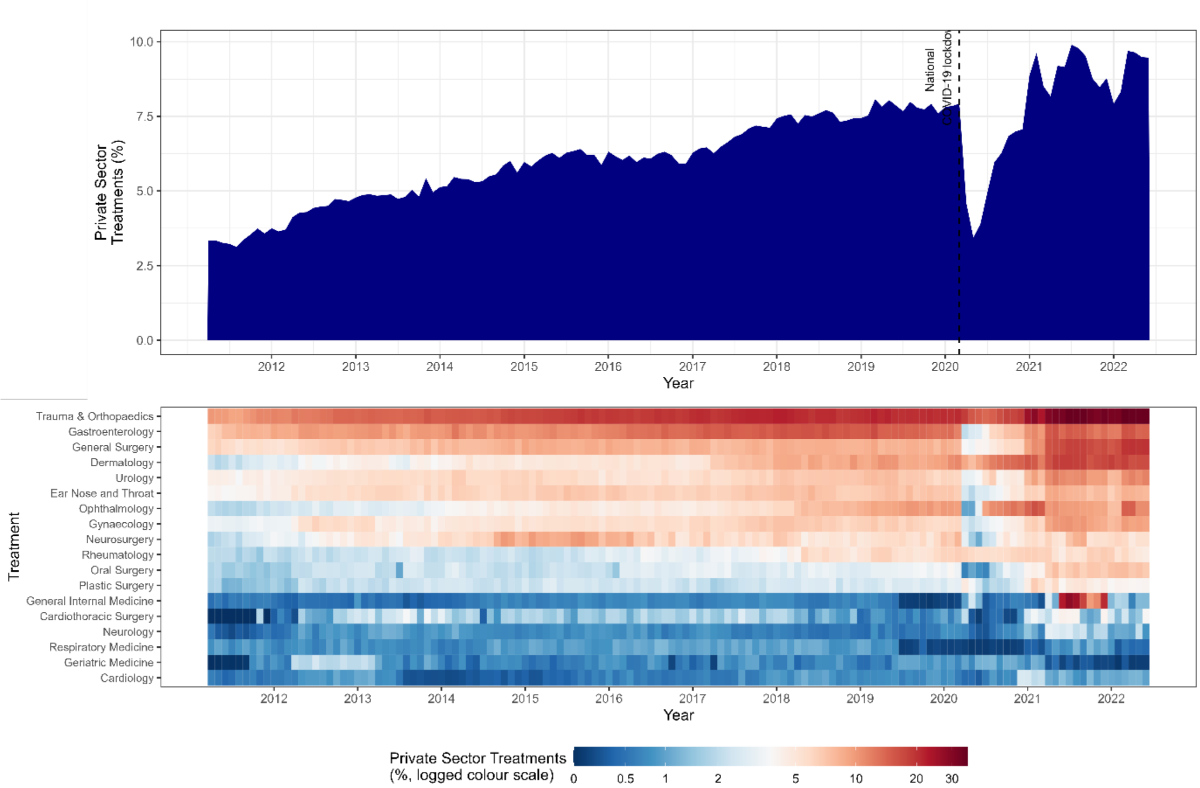

A.9.2 Breakdowns of data by company type

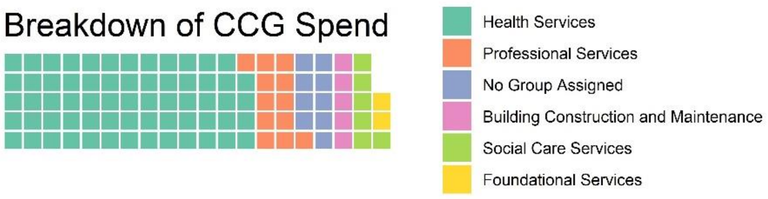

To see a complete list of for-profit companies procured from by CCGs, they are listed at https://raw.githubusercontent.com/BenGoodair/CCG-Outsourcing/main/Data/Companies_House_SIC_lookup.csv

A.10. Test for Multicollinearity

In table 2, we present a regression with all our variables included – here we test for multicollinearity, using VIF. The VIF values are high for a few of the variables – suggesting that some of our control variables may have collinearity – although there is no issue with our main variable of interest (outsourcing %). Part of the reason we run the other models in table 2, is to ensure that separately each control variable is tested.

`lag(Private_Sector_Procurement_Spend)	2.105384
`lag(log(deflated_per_person_benefits))`	1.668254
`lag(log(deflated_per_person_allocation))`	6.883562
`lag(log(deflated_per_person_allocation_la))`	12.041210
total_spend_10millions	13.356740
GDHI_per_person	17.019719
CCGpop	18.351372

References

[Dataset] 1. Rahal C, Knowles I, Barnard S, Mohan J. Introducing NHSSpend: Data and code to parse, harmonize and reconcile NHS procurement data. 2021 [cited 2023 Nov 5]; Available from: https://zenodo.org/records/5054717

[Dataset] 2. Rahal C, Mohan J. The Role of the Third Sector in Public Health Service Provision: Evidence from 25,338 heterogeneous procurement datasets. SocArXiv [Internet]. 2022 Jan 27 [cited 2023 Jan 25]; Available from: https://ideas.repec.org//p/osf/socarx/t4x52.html

[Dataset] 3. NHS England. Statistics » Consultant-led Referral to Treatment Waiting Times [Internet]. 2023 [cited 2023 Jan 25]. Available from: https://www.england.nhs.uk/statistics/statistical-work-areas/rtt-waiting-times/

[Dataset] 4. NHS England. NHS England » Allocations [Internet]. 2023 [cited 2023 Nov 5]. Available from: https://www.england.nhs.uk/allocations/

[Dataset] 5. NHS England. NHS England » Clinical Commissioning Groups' (CCG) accounts (2016-17) [Internet]. 2018 [cited 2023 Nov 5]. Available from: https://www.england.nhs.uk/publication/clinical-commissioning-groups-accounts/

[Dataset] 6. NAO. PCT to CCG (population, ONS 12) [Internet]. 2012 [cited 2023 Nov 5]. Available from: https://www.nao.org.uk/wp-content/uploads/2015/03/Mapping-between-PCTs-and-CCGs-matrices.1.xlsx

[Dataset] 7. ONS. GDP Deflator: Year on Year growth: SA % - Office for National Statistics [Internet]. 2023 [cited 2023 Nov 5]. Available from: https://www.ons.gov.uk/economy/grossdomesticproductgdp/timeseries/ihys/

8. Alexiou A, Mason K, Fahy K, Taylor-Robinson D, Barr B. Assessing the impact of funding cuts to local housing services on drug and alcohol related mortality: a longitudinal study using area-level data in England. International Journal of Housing Policy. 2023 Apr 3;23(2):362–80.

[Dataset] 9. DWP. Households on Universal Credit Dashboard [Internet]. 2023 [cited 2023 Nov 5]. Available from: https://stat-xplore.dwp.gov.uk/webapi/metadata/dashboards/uch/index.html

10. Beatty C, Fothergill S. Hitting the poorest places hardest: The local and regional impact of welfare reform [Internet]. 2016 [cited 2023 Apr 10]. Available from: https://www.shu.ac.uk/centre-regional-economic-social-research/publications/hitting-the-poorest-places-hardest-the-local-and-regional-impact-of-welfare-reform

[Dataset] 11. ONS. Avoidable mortality in the UK - Office for National Statistics [Internet]. 2021 [cited 2023 Feb 28]. Available from: https://www.ons.gov.uk/peoplepopulationandcommunity/healthandsocialcare/causesofdeath/bulletins/avoidablemortalityinenglandandwales/2019
